# Revealing Hi-C subcompartments by imputing inter-chromosomal chromatin interactions

**DOI:** 10.1038/s41467-019-12954-4

**Published:** 2019-11-07

**Authors:** Kyle Xiong, Jian Ma

**Affiliations:** 1 Joint Carnegie Mellon University-University of Pittsburgh Ph.D. Program in Computational Biology, Pittsburgh, PA 15213 USA; 2 Computational Biology Department, School of Computer Science, Carnegie Mellon University, Pittsburgh, PA 15213 USA

**Keywords:** Computational biology and bioinformatics, Epigenomics, Chromatin structure

## Abstract

Higher-order genome organization and its variation in different cellular conditions remain poorly understood. Recent high-coverage genome-wide chromatin interaction mapping using Hi-C has revealed spatial segregation of chromosomes in the human genome into distinct subcompartments. However, subcompartment annotation, which requires Hi-C data with high sequencing coverage, is currently only available in the GM12878 cell line, making it impractical to compare subcompartment patterns across cell types. Here we develop a computational approach, SNIPER (Subcompartment iNference using Imputed Probabilistic ExpRessions), based on denoising autoencoder and multilayer perceptron classifier to infer subcompartments using typical Hi-C datasets with moderate coverage. SNIPER accurately reveals subcompartments using moderate coverage Hi-C datasets and outperforms an existing method that uses epigenomic features in GM12878. We apply SNIPER to eight additional cell lines and find that chromosomal regions with conserved and cell-type specific subcompartment annotations have different patterns of functional genomic features. SNIPER enables the identification of subcompartments without high-coverage Hi-C data and provides insights into the function and mechanisms of spatial genome organization variation across cell types.

## Abstract (web summary)

Genome-wide mapping of chromatin interactions reveals various levels of 3D genome organization. Here, the authors develop SNIPER, a computational method for identifying subcompartments using Hi-C data with moderate coverage.

## Introduction

In humans and other higher eukaryotes, chromosomes are folded and organized in 3D space within the nucleus and different chromosomal loci interact with each other^1–3^. Recent developments in whole-genome mapping of chromatin interactions, such as Hi-C^4,5^, have facilitated the identification of genome-wide chromatin organizations comprehensively, revealing important 3D genome features such as loops^5^, topologically associating domains (TADs)^6–8^, and A/B compartments^4^. Specifically, at megabase resolution, chromosomes are largely segregated into two compartments, A and B^4,9^. Compartment A regions generally contain open and active chromatin, while compartment B regions are mostly transcriptionally repressed. Further analysis showed that these A/B compartment domains can be inferred from epigenetic status including DNA methylation and chromatin accessibility, as well as DNA replication timing^10^. The separations of B and A compartments in the genome also have near identical agreement with lamina associated domains (LADs) and inter-LADs, respectively^11,12^, suggesting that A/B compartments have different spatial positions in the nucleus. More recently, A/B compartment separations have been observed using other genomic and imaging approaches to probe the 3D genome^13–15^.

In Rao et al.^5^, the A/B compartment definitions were greatly enhanced using high-coverage Hi-C data generated from the human lymphoblastoid (GM12878) cell line. Specifically, Rao et al.^5^ identified Hi-C subcompartments that divide A/B compartments into five primary subcompartments: A1, A2, B1, B2, and B3. These Hi-C subcompartments show distinct and more refined associations with various genomic and epigenomic features such as gene expression, active and repressive histone marks, DNA replication timing, and specific subnuclear structures^5^. A more recent study based on the TSA-seq technology further demonstrated that these subcompartments strongly correlate with cytological distance between the chromatin and specific subnuclear structures such as nuclear speckles and nuclear lamina, reflecting the spatial localization of the chromatin in the nucleus^16^. Therefore, the annotation of Hi-C subcompartments could be extremely useful to provide complementary perspective of the 3D genome in terms of its spatial position in cell nucleus and its functional connections.

Hi-C data from GM12878, which has almost 5 billion mapped paired-end read pairs, is the dataset with the highest coverage to allow reliable identification of subcompartments through clustering inter-chromosomal contact matrices. Unfortunately, when the same clustering procedure is applied on lower coverage inter-chromosomal contact maps from most available Hi-C datasets that typically have 400 million to 1 billion mapped read pairs^5^, the inter-chromosomal contact matrices are often too sparse to reveal clear subcompartment patterns. Recently, a neural network based method called MEGABASE was developed to predict Hi-C subcompartment assignments of chromosome regions with 100 kb resolution using many epigenomic signals as features without using Hi-C data^17^. Based on 84 protein-binding and 11 histone mark ChIP-seq datasets in GM12878, MEGABASE was trained to predict the original subcompartment annotations in GM12878 from Rao et al.^5^ with over 60% consistency in each subcompartment compared to the original annotations (except for the B2 subcompartment). However, most cell types do not have as many ChIP-seq datasets as GM12878 does and some histone marks may even exhibit drastically reduced abundance in other cell lines^18^. Therefore, MEGABASE has limited application to most cell types and it is also unclear how MEGABASE would perform in cell types other than GM12878. Indeed, comparing Hi-C subcompartments across different cell types still has not been possible.

Here we develop a computational method called SNIPER, for nuclear genome Subcompartment iNference using Imputed Probabilistic ExpRessions of high-coverage inter-chromosomal Hi-C contacts. We utilize a neural network framework based on a denoising autoencoder^19^ and multi-layer perceptron (MLP) classifier^20^ that uses moderate coverage Hi-C contact maps, which are typically available, to recover high-coverage inter-chromosomal contact maps and predict the subcompartment labels of genomic regions in 100 kb resolution. A recently developed method HiCPlus^21^ used convolutional neural networks^22^ to impute intra-chromosomal chromatin contacts, but as of now there are no methods to directly impute inter-chromosomal contacts. We demonstrate that SNIPER can accurately recover high-coverage inter-chromosomal Hi-C contact maps in GM12878 such that we can reliably annotate subcompartments, and can significantly outperform MEGABASE. We apply SNIPER to additional eight cell lines, including K562, IMR90, HUVEC, HeLa, HMEC, HSPC, T Cells, and HAP1, to reveal Hi-C subcompartment changes across cell types for the first time. We believe that SNIPER is a useful method to offer new perspectives of genome organization changes with respect to Hi-C subcompartments in different cell types. Our results can also facilitate future work to search for molecular determinants that modulate compartmentalization in different cellular conditions. The source code of SNIPER can be accessed at: https://github.com/ma-compbio/SNIPER.

## Results

### Overview of SNIPER

**Fig. 1:**
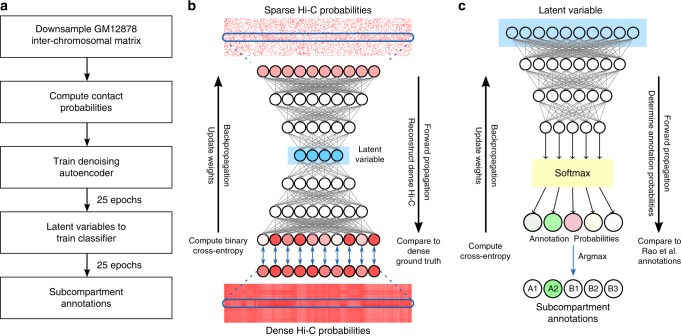
Overview of SNIPER. **a** Flowchart of SNIPER’s training procedure. **b** SNIPER denoising autoencoder. Rows of the low coverage Hi-C probability map are used in the input layer. Weights are optimized using binary cross-entropy (BCE) loss between the reconstructed and ground truth contact probabilities. **c** SNIPER neural network classifier is trained using latent variables from **b** as input and optimized using cross-entropy between predictions and the original annotations based on the high-coverage Hi-C data in Rao et al.^5^

The overall goal of SNIPER is to use only moderate coverage Hi-C data (e.g., approx. 500 million mapped read pairs with approx. 50–70 million inter-chromosomal read pairs) as input to infer subcompartment annotations (Fig. 1). Rao et al.^5^ originally defined subcompartments by using the inter-chromosomal Hi-C matrix from GM12878, constructed from Hi-C contacts between odd-numbered chromosomes along the rows and even-numbered chromosomes along the columns. The authors used a Gaussian hidden Markov model (HMM) to cluster on the rows of the inter-chromosomal matrix. Loci in odd-numbered chromosomes were assigned to five clusters corresponding to the five primary subcompartments. Clusters were separated into A1, A2, B1, B2, or B3 subcompartments based on the Spearman correlations between clusters. To define subcompartments in even-numbered chromosomes, Rao et al.^5^ applied the clustering method to the transpose of the inter-chromosomal matrix.

The SNIPER framework is comprised of two separate neural networks, a denoising autoencoder^19^ (Fig. 1b) and a MLP classifier (Fig. 1c). The autoencoder takes as inputs rows in a sparse inter-chromosomal Hi-C matrix (in 100 kb resolution) for genomic regions in odd-numbered chromosomes along the rows and regions in even-numbered chromosomes along the columns. The autoencoder outputs dense contacts between a given region in odd-numbered chromosomes and all regions in even-numbered chromosomes. At the same time, its encoder outputs low-dimensional latent variables that represent features in the sparse matrix which capture dense chromatin contacts (Fig. 1b). The latent variable compresses high-dimensional genome-wide inter-chromosomal contacts of each genomic region into a much lower dimension, and is subsequently input into the classifier that categorizes the regions into one of five primary subcompartment classes—A1, A2, B1, B2, and B3 (based on GM12878 annotations) (Fig. 1c). Note that although Rao et al.^5^ defined an additional B4 subcompartment, it is only present and specifically defined in chromosome 19, occupying less than 0.4% of the genome. We therefore did not train SNIPER to consider B4. We then train a separate autoencoder and classifier to annotate regions in even-numbered chromosomes. We convert Hi-C contacts into contact probabilities to mitigate the effects of extreme Hi-C signals (see Methods section). By using low dimensional representations of complex genome-wide chromatin contacts, we can predict subcompartment annotations using a basic multi-layer perceptron network. A detailed description of SNIPER is provided in the Methods section.

Note that GM12878 has very high Hi-C coverage (approx. 5 billion mapped read pairs genome-wide with 740 million inter-chromosomal read pairs between the odd-numbered chromosomes and the even-numbered chromosomes) while other cell types typically have just a few hundred million read pairs genome wide with less than 100 million inter-chromosomal read pairs. To reflect coverage in other cells types, we downsampled the GM12878 Hi-C dataset to around 500 million read pairs genome wide by randomly removing 90% (or 95%) of its original reads, resulting in about 74 million (or 37 million) inter-chromosomal read pairs between the odd chromosomes and the even chromosomes. The inter-chromosomal Hi-C matrix from the downsampled GM12878 data is then used to train the autoencoder. Hi-C data of lower coverage cell lines can then be input into the trained networks to infer their dense Hi-C matrices and subcompartment annotations.

### SNIPER can accurately predict Hi-C subcompartments in GM12878

We first evaluated the performance of SNIPER in inferring subcompartments in GM12878 using downsampled Hi-C data because the annotation based on high-coverage Hi-C is readily available from Rao et al.^5^. We use confusion matrices to assess the overall accuracy of SNIPER compared to Rao et al.^5^ annotations in GM12878 and also show performance differences in different subcompartments. We define accuracy as the fraction of 100 kb chromatin regions whose SNIPER annotations match Rao et al.^5^ annotations. The neural networks in SNIPER expect inputs with the same length, but the inter-chromosomal Hi-C matrix is not symmetric. We cannot simply transpose the matrix and use a single SNIPER model to predict subcompartment annotations in both odd and even-numbered chromosomes. We therefore trained two separate models of SNIPER, one to classify subcompartments in odd-numbered chromosomes, and one for predictions in even-numbered chromosomes. The odd-numbered chromosome model is trained on loci from chromosomes 1, 3, 5, and 7 and tested on loci in the remaining odd-numbered chromosomes. Similarly, the even-numbered chromosome model is trained using chromosomes 2, 4, 6, 8, and 10 and tested on the remaining even-numbered chromosomes.

**Fig. 2:**
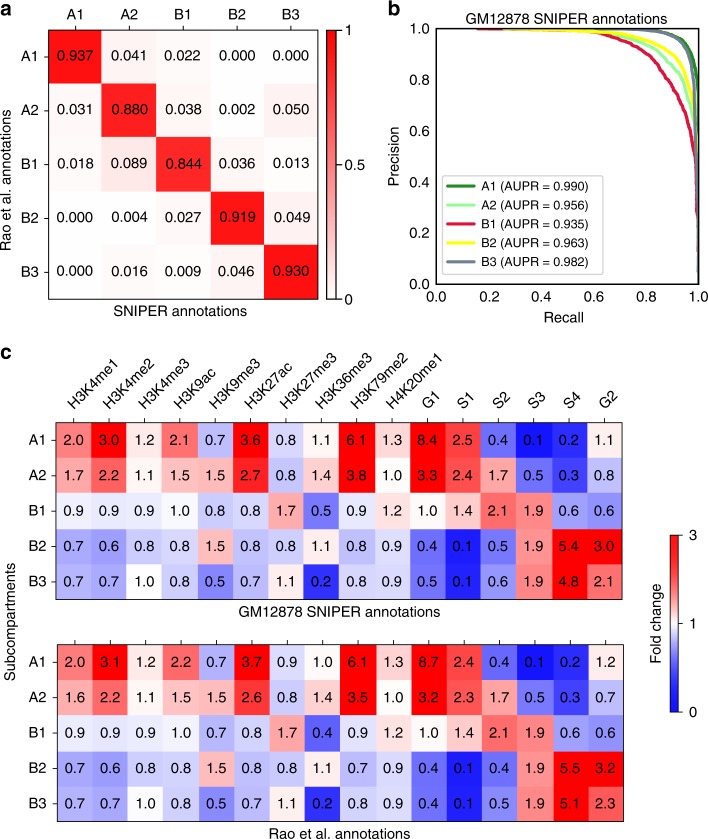
SNIPER performance in GM12878. **a** Confusion matrix between SNIPER predictions and the original subcompartment annotation based on the high-coverage Hi-C in Rao et al.^5^. **b** Precision-recall curve and AUPR values for the prediction of each subcompartment. **c** Histone mark and replication timing fold change profiles constructed for SNIPER results (top) and the Gaussian HMM subcompartment calls based on the full dataset^5^ (bottom). Fold change of an epigenetic mark in each subcompartment is defined as the median signal of the mark divided by the median signal across all subcompartments. Source data are available in the Source Data file

The SNIPER annotations of A1, A2, B1, B2, and B3 for each 100 kb genomic region in GM12878 match 93.7%, 88.0%, 84.4%, 91.9%, and 93.0% of Rao et al.^5^ subcompartment annotations, respectively (Fig. 2a). Using chromosomes 9, 11, 13, 15, 17, 19, and 21 as the training set for the odd-numbered chromosome model, SNIPER achieves similarly high accuracy (Supplementary Fig. 1). The average precision of SNIPER predictions in each subcompartment also remains high, with areas under the precision-recall curve (AUPR) of 0.990, 0.956, 0.935, 0.963, and 0.982, respectively (Fig. 2b). In addition, in 10-fold cross validation, the accuracy of SNIPER remains high with low variance among training folds (Table 1). Latent variables for all chromatin regions are divided into 10 partitions, each of which achieves similar accuracy as compared to Rao et al.^5^ annotations (Supplementary Table 1).

**Table 1:** Accuracy of predicting GM12878 annotations for 100 kb bins using the baseline Gaussian HMM, MEGABASE^17^, and SNIPER (average across 10-fold cross validation)

	Accuracy				
Method	A1	A2	B1	B2	B3
Gaussian HMM	0.918	0.776	0.681	0.638	0.767
MEGABASE	0.728	0.718	0.614	0.196	0.831
SNIPER (10-Fold CV)	0.960	0.912	0.861	0.874	0.959
	Variance				
SNIPER	4.59E−04	5.28E−04	3.90E−04	1.08E−03	2.17E−04

Importantly, we found that SNIPER outperforms the baseline Gaussian HMM and the recently published MEGABASE by using the subcompartment annotations from Gaussian HMM based on the full inter-chromosomal Hi-C matrix^5^ as a benchmark (Fig. 2a, Supplementary Figs. 2A,B). Table 1 shows that SNIPER significantly outperforms MEGABASE and Gaussian HMM in all subcompartments. Most notably, SNIPER accurately annotates B2 and B3 regions, whereas MEGABASE frequently confuses B2 and B3 (Supplementary Fig. 2b). We also computed the AUPR of the Gaussian HMM model (Supplementary Fig. 3), which is much worse than the AUPR from SNIPER for all subcompartments (as compared to Fig. 2b).

We then extended the evaluation by comparing SNIPER and Gaussian HMM at different read coverage levels based on different inter-chromosomal read pairs as compared to the full coverage inter-chromosomal contact map where there are 740 million read pairs between the odd-numbered and the even-numbered chromosomes. In Table 2, we show the percentage of SNIPER and Gaussian HMM annotations at various coverage levels that match the reference annotations. SNIPER accurately predicts subcompartment annotations with more stable performance at a wide range of coverage levels. SNIPER outperforms Gaussian HMM when coverage is between 2% and 60% of the original inter-chromosomal contacts and significantly outperforms Gaussian HMM when coverage is at 10% of the original contacts or lower. Most of the available Hi-C datasets, including the non-GM12878 cell lines in this study, typically has around 10% of the full coverage of GM12878 (Supplementary Table 2). This evaluation demonstrates the stable performance of SNIPER, which is significantly better than Gaussian HMM when the Hi-C data coverage is moderate or low.

**Table 2:** Subcompartment prediction accuracy of the Gaussian HMM and SNIPER at various levels of coverage. SNIPER outperforms the Gaussian HMM when the inter-chromosomal matrix is below 60% of the original GM12878 inter-chromosomal read count and the performance is dramatically better at lower coverage levels

Prediction accuracy at different coverage levels					
Coverage${}^{\ddagger }$	14.8 (2%)	22.2 (3%)	29.6 (4%)	37 (5%)	74 (10%)
Gaussian HMM	46.46%	50.66%	56.51%	57.27%	65.04%
SNIPER	88.91%	88.83%	90.08%	91.05%	92.69%
Coverage	148 (20%)	296 (40%)	444 (60%)	592 (80%)	740 (100%)
Gaussian HMM	88.28%	93.64%	95.85%	97.09%	99.82%
SNIPER	93.71%	94.12%	91.91%	87.69%	84.07%

${}^{\ddagger }$The first number refers to the inter-chromosomal coverage in million read pairs (between the odd-numbered chromosomes and the even-numbered chromosomes) and the number in the parenthesis is the percentage as compared to the full dataset

We then compared the prediction from SNIPER with histone mark ChIP-seq and DNA replication timing data in GM12878 (Fig. 2c) obtained from the ENCODE project^23^. We determined enrichment of different functional genomic signals in each SNIPER subcompartment and Rao et al.^5^ subcompartment by following the same procedure in the Supplement Section V.b.2 from Rao et al.^5^. We also compared the epigenetic mark enrichment at genomic regions where subcompartment annotations from Gaussian HMM and SNIPER trained on low coverage GM12878 differ from the reference annotations obtained from fitting a Gaussian HMM on GM12878 with full coverage (Supplementary Fig. 4). Among such regions, we found that SNIPER predictions retain more agreement (as compared to Gaussian HMM predictions) with the reference annotations in terms of enrichment of functional genomic signals. Overall, we found that the enrichments with histone marks and replication timing are very consistent with the results in Rao et al.^5^ (Fig. 2c). This further suggests the overall high concordance between the predictions from SNIPER, which only uses downsampled data (10% of the original read pairs), and the Hi-C subcompartment annotations based on the full dataset from Rao et al.^5^.

### SNIPER annotations in other cell types are supported by genomic and epigenomic data

**Fig. 3:**
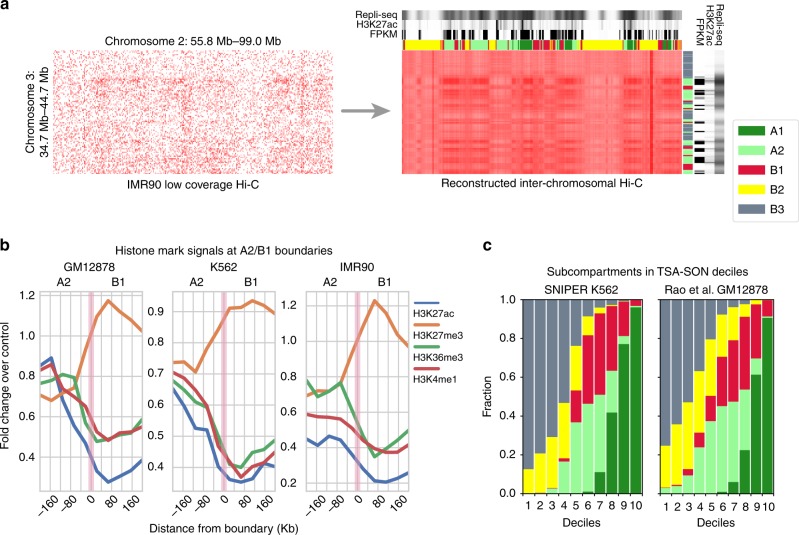
SNIPER predictions correlate with various functional genomic data. **a** Reconstruction of the inter-chromosomal Hi-C contact matrix in IMR90. This example between chromosomes 2 and 3 shows that SNIPER imputes missing contacts in the sparse matrix, recovers subcompartment-specific contact patterns, and predicts annotations that correlate with DNA replication timing Repli-seq, H3K27ac ChIP-seq, and RNA-seq (FPKM). **b** Normalized histone mark signal changes at the boundary between A2 (left) and B1 (right) in GM12878, IMR90, and K562. **c** Subcompartment distribution in K562 SON TSA-seq deciles for the SNIPER K562 subcompartments (left) and the Rao et al.^5^ GM12878 subcompartments (right). Source data are available in the Source Data file

Because subcompartment annotations from high-coverage Hi-C data are only available in GM12878, we cannot directly compare the SNIPER predicted subcompartments in other cell types to the results based on high-coverage Hi-C. We therefore specifically focused on functional genomic data in K562 and IMR90, where a large number of epigenomic datasets are available, to evaluate SNIPER predictions. Fig. 3a is an example showing that SNIPER recovered missing contacts from the sparse low coverage inter-chromosomal contact map of IMR90, revealing much clearer compartmentalized contact patterns whose boundaries strongly correlate with shifts in functional genomic data. A1 and A2 regions generally have early replication timing and dense H3K27ac and RNA-seq signals, whereas regions in B1, B2, and B3 replicate later and have lower transcriptional activities (see Supplementary Fig. 5 and Supplementary Fig. 6). The patterns of functional genomic signal enrichment in different subcompartments are similar to what we observed from GM12878 (Fig. 2c and Supplementary Fig. 7). We also compared the subcompartments with chromatin state annotated by ChromHMM^24^ and Segway-GBR^25^, respectively, in multiple cell types. We further confirmed the generally consistent patterns between subcompartments and active/repressive chromatin states (Supplementary Notes, Supplementary Fig. 8, and Supplementary Fig. 9).

In addition, we observed significant shifts of histone mark signals in 400 kb neighborhoods around subcompartment boundaries between A2 and B1 (Fig. 3b for results in GM12878, K562, and IMR90) and other subcompartment boundaries (see Supplementary Fig. 10). Supplementary Fig. 11 shows the total number of transitions between predicted subcompartments observed in genomic regions for each of the nine cell types that SNIPER was applied to. We focus on A2 to B1 transitions here as they are among the most frequent in the cell types in our analysis, e.g., about six times more frequent than A1 to B1 transitions. Furthermore, A2 and B1 are associated with euchromatin and facultative heterochromatin, respectively^5^, and can both be relatively transcriptionally active. As a result, the large shift in histone mark signals across their boundaries signifies SNIPER’s ability to differentiate spatially close and functionally similar subcompartments. Active marks such as H3K9ac, H3K27ac, and H3K36me3 are generally more enriched in A2 than B1 with a dramatic drop moving across the boundary, consistent with the significantly lower enrichment of active marks in B1 (see Rao et al.^5^; Fig. 2d), whereas the facultative heterochromatin mark H3K27me3 becomes more enriched across the A2-B1 boundary. These patterns of changes in epigenomic signals at the boundaries of subcompartments are consistent with changes in histone mark signals at subcompartment boundaries shown by Rao et al.^5^ and more recently by Chen et al.^16^, and the average log ratio between two epigenomic signals shown by Robson et al.^26^. We also observed changes of histone mark signals around A2 and B1 boundaries in downsampled GM12878, K562, and IMR90 using subcompartment annotations from Gaussian HMM clustering (Supplementary Fig. 12). Compared to what we observe from the predictions based on SNIPER, the patterns of signal changes around A2 and B1 boundaries in GM12878 and IMR90 based on Gaussian HMM are similar. However, signals in K562 annotated by Gaussian HMM showed little difference around A2 and B1 boundaries. This observation suggests that Gaussian HMM may not be appropriate to identify subcompartments with accurate boundaries for all cell types.

We found that genomic regions replicate much earlier in A1 and A2 subcompartments than in B subcompartments (Supplementary Fig. 13) in GM12878, K562, and IMR90. In addition, it is known that the level of histone modification of H3K27ac is associated with enhancer activities^27^ and sometimes also transcriptionally active inter-LADs^12^. We found that H3K27ac generally has much higher signal in predicted A1 and A2 than in B compartment regions, and is virtually absent in predicted B2 and B3 regions (Supplementary Fig. 13). Higher H3K27ac signals in B1-annotated regions suggest less transcriptional activity than regions in A1 and A2 but more activity than B2 and B3. Intermediate levels of transcriptional activity and increased abundance of H3K27me3 in the predicted B1 regions are indicative of its association with facultative heterochromatin^12^ (see Supplementary Fig. 13 that shows consistent patterns across cell types).

The recently developed TSA-seq can reveal cytological distance between chromosomal regions to specific subnuclear structures^16^. TSA-seq is a new genome-wide mapping method that provides cytological distance between subnuclear structures with proteins of interest and chromatin regions. For example, the SON protein is preferentially localized at nuclear speckles and lamin B1 is a major nuclear lamina protein component. In Chen et al.^16^, it was reported that SON TSA-seq (which targets nuclear speckles) showed that transcription hot zones (peaks in SON TSA-seq) are primarily associated with the A1 subcompartments (mostly for peak summit of high peaks) and A2 subcompartments (mostly for smaller peaks with lower SON TSA-seq scores). However, the caveat of the comparison in Chen et al.^16^ is that the TSA-seq data are based on K562 cells but the subcompartment annotations used are in GM12878 from Rao et al.^5^.

We therefore directly compared SNIPER annotations in K562 with TSA-seq scores (Fig. 3c) to demonstrate the benefit of having subcompartment annotations in K562. We used SON and LaminB TSA-seq data that measure the distance to nuclear speckles and nuclear lamina in K562, respectively (note that TSA-seq is not available for other cell types studied in this work). We found that SON TSA-seq signals show significant stratification of the predicted subcompartments in K562 with more consistency. Importantly, we further confirmed the observations from Chen et al.^16^ that the highest SON TSA-seq peaks are associated with A1 subcompartments and A2 subcompartments are also close to the nuclear interior with relatively high SON TSA-seq scores. In particular, the highest SON TSA-seq decile is almost exclusively associated with A1 regions in the SNIPER annotations. In contrast, using Rao et al.^5^ GM12878 annotations, a significantly higher portion of the highest SON TSA-seq decile is associated with B1 regions. 71.65% of the 3 highest SON TSA-seq deciles are labeled as A1 using the K562 SNIPER annotations, whereas 54.10% are labeled as A1 when using the GM12878 Gaussian HMM annotations. Therefore, the K562 SNIPER annotations made the observations from Chen et al.^16^ even clearer, namely the highest deciles of SON TSA-seq strongly correlate with A1 subcompartments. Furthermore, virtually none of the SNIPER predicted A2 regions are binned into the lowest 2 deciles while the Rao et al.^5^ A2 regions are present in all low deciles. A scatter plot of SON TSA-seq and LaminB TSA-seq percentiles (Supplementary Fig. 14) shows that almost all subcompartments tend to cluster better based on SNIPER K562 subcompartment annotations instead of the original GM12878 subcompartment annotations. We found that in general SNIPER annotations in K562 are partitioned into subcompartments with narrower SON TSA-seq and LaminB TSA-seq signal ranges as compared to Rao et al.^5^ annotations in GM12878. These results suggest that the SNIPER subcompartment annotations in K562 are accurate and offer a more direct comparison with SON and LaminB TSA-seq in K562 than the Rao et al.^5^ GM12878 subcompartment annotation (which was the approach Chen et al.^16^ used).

### SNIPER facilitates the identification of subcompartment patterns across different cell types

**Fig. 4:**
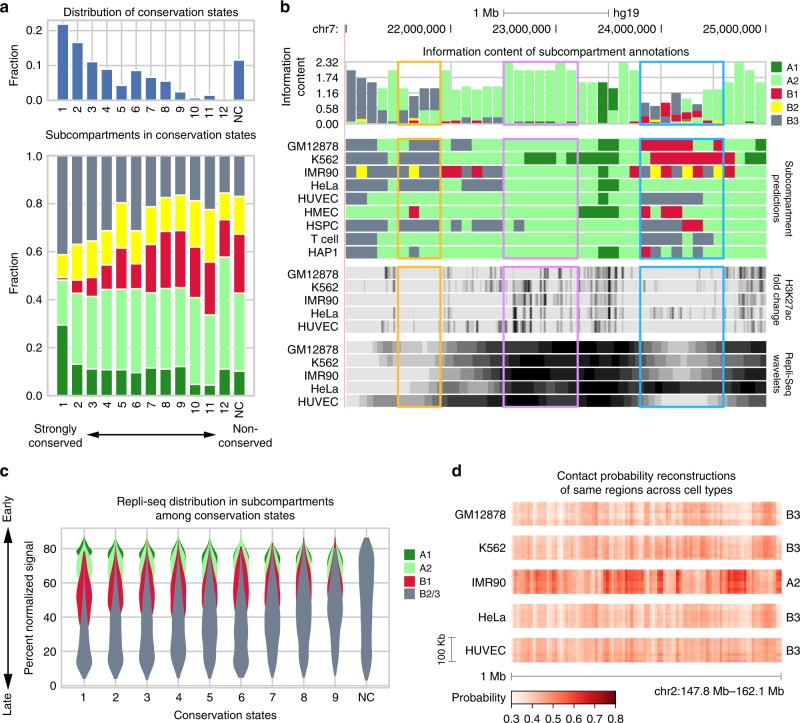
SNIPER allows comparisons of subcompartments across different cell types. **a** (Top) Distribution of thirteen conservation states in the genome. (Bottom) Distribution of subcompartment regions in each conservation state. **b** UCSC Genome Browser shot displaying the information content (IC), cross cell type predictions, H3K27ac fold change, and smoothed Repli-seq wavelets of each 100 kb region. Subcompartments offering the most information, associated with taller bars in the IC track, are more conserved across nine cell types. Highlighted color boxes show three regions with distinct patterns across cell types. **c** For each conservation state, we show Repli-seq signal distribution in the most frequent subcompartment annotations among nine cell types at each 100 kb region. Regions in conservation state 1 are the most conserved, reflected by low variance of Repli-seq signal in each subcompartment. Regions in the NC state are the most dynamic, suggesting multiple annotations for a single chromatin region and high variance in Repli-seq signal. **d** Hi-C reconstructions across cell types in chr18 (47.1–47.9 Mb) where IMR90 is A2-specific and other cell lines are predicted as B3. Source data are available in the Source Data file

We next applied SNIPER to predict Hi-C subcompartments in K562, IMR90, HeLa, HUVEC, HMEC, HSPC, T Cells, and HAP1 (see distributions of subcompartments in all cell types in Supplementary Fig. 15). Note that not all cell types have the same level of Hi-C coverage (see Supplementary Table 2). We applied the SNIPER model trained on 10% of GM12878’s original read pairs to K562, IMR90, HSPC, and T cells and the SNIPER model trained on 5% of the original read pairs to HeLa, HUVEC, HMEC, and HAP1. Together with the subcompartments in GM12878, this allows us to perform a detailed comparison of subcompartment conservation and changes across multiple cell types. 100kb genomic regions are partitioned into 13 conservation states (see Methods section for detailed definitions) based on the subcompartment annotation distribution of each region among nine cell types. States are termed states 1–12 and NC, sorted by ascending entropy of cross cell type annotations, of which state 1 has the lowest entropy and refers to genomic regions with the most conserved cross cell type annotations, states 2–12 gradually increase in entropy and decrease in conservation, and state NC refers to the dynamic non-conserved state. States 1–3 occupy large fractions of the genome, indicating that a large portion of the genome contains relatively conserved subcompartment annotations (Fig. 4a). Notably, the A1 and B3 subcompartments appear to be the most conserved subcompartments across cell types, with about 30% and 40% representation in the most conserved state 1, respectively. By contrast, there is less B1 presence in the more conserved states, consistent with the observations in Rao et al.^5^ that B1 is associated with facultative heterochromatin. The NC state comprised about 11% of the genome and contained relatively few A1 and B3 regions, suggesting A1 and B3 are likely more conserved across cell types than other subcompartments.

Information content (see Methods section), H3K27ac ChIP-seq fold change, and smoothed Repli-seq signals at each region of the Genome Browser shot (Fig. 4b) show conserved and dynamic functional genomic patterns across cell types. Similar to information content of position weight matrices for transcription factor binding motifs, subcompartment information content reflects the information gained from annotations across all cell types in 100kb genomic bins. Genomic regions with high information content have significantly more conserved annotations across cell types than regions with low information content. Conserved A2 regions, shown in the purple segment of Fig. 4b, can be expected to retain conserved annotations and functional genomic patterns even in cell lines not in our analysis. Less informative regions (Fig. 4b, yellow and blue) exhibit inconsistent functional genomic signals. Regions with HeLa-specific A2 annotations (Fig. 4b, blue rectangle) show increased abundance of H3K27ac signal and much earlier replication timing compared to other cell lines. These regions are annotated as B1, B2, and B3 in other cell types and correspond to lower H3K27ac signals and later replication timing.

The amount of information gained in each conservation state is reflected in the Repli-seq distribution in subcompartment modes across states (Fig. 4c). For each region in a conservation state, its cross cell type Repli-seq signals were binned according to the region’s mode, defined as the most frequent subcompartment annotation among 9 cell types. We then plotted the violin plots of Repli-seq signals in each mode of the conservation state. We binned Repli-seq signals for all other conservation states except states 10, 11, and 12, which contained too few 100 kb regions. We found that more conserved states show less variance of Repli-seq signals in each mode because cross cell type predictions are less varied. Less conserved states such as states 8 and 9 exhibit much higher Repli-seq variance in each mode, especially B1 and B3. Repli-seq distributions of all modes virtually overlap in the NC state, further showing high variance of functional genomic signals in more dynamic subcompartment regions across cell types. Because Repli-seq is virtually identically distributed in B2 and B3, the two subcompartments are merged in Fig. 4c. In addition, we found strong correlation between the subcompartments conservation states defined by SNIPER across cell types and the constitutive early, constitutive late, and developmentally regulated replication timing domains during ES cell differentiation^28^ (Supplementary Notes and Supplementary Fig. 16).

Hi-C reconstructions at genomic regions with cell-type specific annotations are distinct from the same regions in other cell types. Fig. 4d shows an example of A2 regions specific to IMR90 that exhibit significantly more frequent contacts compared to the same region in other cell types, which are annotated as B3. Taken together, these results demonstrate that SNIPER provides us with the capability to reliably compare Hi-C subcompartment annotations in multiple cell types and reveal cross cell type patterns of conservation and variation of Hi-C subcompartments.

### Conserved and cell-type specific subcompartment patterns show distinct gene functions

Genomic regions where transcription activity is high in a single cell type may reveal genes that contribute to unique cellular functions. Here, we focus on both cell-type specific A1 subcompartments and A2 subcompartments, both of which tend to have more genes with high transcriptional activity. We compiled cell-type specific subcompartment annotations across GM12878, K562, IMR90, HeLa, HUVEC, HMEC, and T Cells and the gene expression profiles. We identified genes in each cell type that are significantly expressed (see Methods section) and also belong to any region with a cell-type specific subcompartment annotation. A background of all annotated human genes in Ensembl V95 is used. Cell-type specific subcompartment annotations can provide a general picture of functions enriched in specific cell types such as GM12878, K562, HMEC, and T cells (Supplementary Table 4). Specifically, we show that significantly expressed genes are associated with immunoglobulin and B cell differentiation in GM12878, anemia and abnormal hemoglobin in K562, keratinization and the cornified envelope in HMEC, and a variety of immune system processes in T cells. However, genes in cell-type specific A1 and A2 subcompartments in IMR90, HeLa, and HUVEC do not necessarily produce enrichment that explains cell-type specific functions. We observe that when we use significantly expressed genes in A1 and A2 individually in GO analysis, the enriched terms are similar but some cell-type specific functions are absent from the results. In addition, as a contrast, we show that the cell-type specific functional enrichments are weaker when we do not use cell-type specific A1 and A2 subcompartment annotations (Supplementary Notes and Supplementary Table 5). We also found that the subcompartments identified by SNIPER reveal much clearer cell-type specific functions as compared to the Gaussian HMM annotations (Supplementary Notes and Supplementary Table 6). These results suggest that cell-type specific subcompartment annotations from SNIPER show cell-type function. However, a single cell-type specific region may contain a small number of key genes most strongly associated with such spatial chromatin localization changes and many genes that act as passengers that obscure distinct functions and pathways.

GO terms associated with constitutive A1 and A2 subcompartment annotations may reveal housekeeping processes required for basic cellular functions. In Supplementary Table 4 (bottom), the most enriched biological processes from GO enrichment analysis are related to metabolic processes. The Bonferroni-corrected *p*-values of these enriched processes show that genes in constitutive A1 and A2 regions contain significant housekeeping functions, which play distinct roles as compared to genes in cell-type specific subcompartments.

### Cross-cell type comparison reveals histone marks and TF motifs important for subcompartment changes

Our earlier analysis showed that various types of histone marks are associated with subcompartments, which are consistent with the original observations in Rao et al.^5^. Di Pierro et al.^17^ later used 11 histone marks to predict subcompartments in GM12878 (based on the reduced MEGABASE model). However, the connections between histone mark variations and subcompartment changes in different cell types remain unclear. The SNIPER annotations gave us the opportunity to study this across cell types.

Among the cell types that we applied SNIPER to, GM12878, K562, IMR90, HeLa, HUVEC, and HMEC share histone mark ChIP-seq data, including H3K27ac, H3K27me3, H3K36me3, H3K4me1, H3K4me2, H3K4me3, H3K79me2, H3K9ac, H3K9ac, and H4K20me1. We trained a Random Forest (RF) classifier from scikit-learn^29^ based on histone marks to discriminate between cell-type specific B2 and B3, A1 and A2, and B1 and A2, given that these are the subcompartment changes with more subtle differences. An example of a chromatin region with a cell-type specific subcompartment annotation is illustrated in Supplementary Fig. 17.

For each region with cell-type specific subcompartment annotations, we compiled the histone marks of the cell type whose annotation is different from all other cell types. We used these histone marks as the input to the RF classifier (see Supplementary Methods). We identified genomic regions annotated as B2 or B3 in one cell type and annotated as B3 or B2, respectively, in all other cell types. After training, the RF classifier is able to distinguish between cell-type specific B2 and B3 regions in the test set with an average of 83.94% accuracy over 10 training runs. This is much higher than the accuracy MEGABASE achieved when using 11 histone marks to discriminate between B2 and B3, as shown in Fig. S29 of Di Pierro et al.^17^. The classifier consistently found that the most important histone marks that delineate between B2 and B3 are H3K4me3, H3K36me3, and H3K27ac. Next we re-trained the RF classifier to distinguish between cell-type specific A1 and A2, and between cell-type specific A2 and B1, respectively, achieving an average accuracy of 85.34% and 84.19%, respectively. The most important feature for distinguishing A1 and A2 is H3K36me3 followed by H4K20me1 and H3K9me3. The most important histone marks for separating A2 and B1 include H3K36me3, H3K79me2, and H4K20me1.

In addition, we explored whether there are transcription factor (TF) binding motifs enriched in cell-type specific subcompartments, which indicate possible roles of certain TF regulators in modulating subcompartment change globally. Here we focused on cell-type specific A2 subcompartments as a proof-of-principle. We scanned motifs using FIMO^30^ based on the JASPAR^31^ database to determine the most enriched TF motifs in each cell type (see Supplementary Methods). All enriched motifs with Bonferroni-corrected *p*-values less than 0.05 are sorted by each motif’s fold change over its background frequency. We define fold change as the frequency of the motif in DNase-seq peaks in cell-type specific A2 regions divided by the frequency of the motif in DNase-seq peaks in all regions (Supplementary Methods). The motifs with the 20 highest fold change in each cell type are shown in Supplementary Fig. 18. Notably, TFAP2B and TFAP2C, which belong to the activating enhancer-binding protein family, are enriched in cell-type specific A2 regions in K562, HeLa, HUVEC, and HMEC. These enriched TF motifs suggest that the corresponding TFs in different cell types may play roles in cooperatively modulating global spatial localization of the A2 subcompartment.

Taken together, the SNIPER annotations facilitate the identification of important histone marks and TF regulators associated with cell-type specific subcompartment changes globally. These results may pave the way for further experimental validations to identify possible molecular determinants and sequence-level features that modulate compartmentalization.

## Discussion

In this work, we introduced SNIPER, a computational method that imputes inter-chromosomal contacts missing from sparse Hi-C datasets and predicts subcompartment annotations at 100kb scale across multiple cell types. We found that SNIPER annotated subcompartments in the GM12878 with high accuracy and outperformed a state-of-the-art method, MEGABASE. In GM12878, K562, IMR90, HeLa, HUVEC, HMEC, HSPC, T cells, and HAP1, we showed that SNIPER predictions correlate well with functional genomic data including histone marks, replication timing, RNA-seq, and TSA-seq. Genomic regions with conserved SNIPER annotations across these nine cell types occupy a significant portion of the genome (21.87%) and shared similar abundance of epigenomic signals. Regions with constitutive A1/A2 annotations are generally associated with housekeeping functions and pathways. Cell-type specific A1/A2 annotations correlate with biological processes specific to some cell types.

SNIPER is able to achieve accurate subcompartment annotations in cell types with inter-chromosomal Hi-C coverage as low as 15 million read pairs. In this study, Hi-C data for different cell types typically have more than 15 million inter-chromosomal Hi-C read pairs, between 50 million and 110 million (Supplementary Table 2, suggesting that the SNIPER subcompartment predictions are accurate. Compared to GM12878 annotations in Rao et al.^5^, we only need approximately 50 times fewer Hi-C reads to reliably annotate subcompartments using SNIPER. Therefore, SNIPER has the potential to significantly reduce the cost of Hi-C experiments to analyze subcompartments across many different cellular conditions.

The Hi-C subcompartment predictions from SNIPER can be compared to results based on other analysis approaches and datasets. For example, we expect that the SNIPER predictions of Hi-C subcompartments can be used to further validate and compare with results from polymer simulations^32,33^, 3D genome structure population modeling^34,35^, and regulatory communities mining based on whole-genome chromatin interactome and other molecular interactomes in the nucleus^36,37^. In addition, recently published new genome-wide mapping methods^13,14,16^ may provide additional training data other than Hi-C, as well as experimental data validation to improve our method.

Currently SNIPER is limited by its training data, which contains a small fraction of the inter-chromosomal mapped read pairs from the original Hi-C reads in GM12878. The Hi-C coverage of other cell lines tends to vary, which can impact the overall accuracy when applied to some cell lines. As a result, SNIPER could incorrectly annotate some regions in a cell line if Hi-C coverage is too high or too low, although our extensive evaluation reveals that the performance of SNIPER is highly stable (Table 2). Large-scale structural variations in the genome may also have an impact on subcompartments but our analysis based on the cancer cell lines K562 and HeLa in this work suggests that the impact may not be significant (see Supplementary Fig. 7 which shows similar enrichment patterns of functional genomic data as compared to GM12878). We believe that this is mainly due to the robustness of the autoencoder embedding of inter-chromosomal contact matrices used in SNIPER. However, further work is required to study the effects of structural variations and copy number alterations on specific loci in terms of subcompartment changes, which may be of particular importance in analyzing cancer genomes. In addition, the ratio between intra-chromosomal and inter-chromosomal reads can vary across cell lines, which we did not explicitly control for. This ratio could exhibit high variance across different cell types and influence the accuracy. Future work should make SNIPER more coverage invariant and produce consistent annotations regardless of the Hi-C coverage of its inputs.

The Hi-C subcompartment annotations used in SNIPER largely rely on the original annotations in GM12878 from in situ Hi-C from Rao et al.^5^. It remains to be explored to improve SNIPER for a wider range of experimental protocols for chromosome conformation. Although the results in this work demonstrate that these subcompartment definitions may well represent primary subcompartments in many cell types, it is also possible that some cell types may have their distinct subcompartment organizations. Future work can be performed to train SNIPER to categorize genomic regions into different sets of subcompartments not limited to the five primary subcompartments used in this work. We made initial efforts to train a SNIPER model based on wild-type HCT116 and apply to cohesin-depleted HCT116 based on the Hi-C data from Rao et al.^38^, demonstrating the capability of SNIPER to analyze cell types whose Hi-C contact patterns are more different than GM12878 (see Supplementary Notes and Supplementary Fig. 19 and Supplementary Fig. 20).

From the GO analysis, the inability to reveal cell-type specific functions in some cell types suggests more work should be done to determine important genes in facultative subcompartment regions that are most significantly associated with cell-type specific spatial localization and function. It is indeed a challenging and intriguing question to search for the molecular determinants that modulate changes in compartmentalization as many genes are correlated with each other in the entire subcompartment domain. Earlier work identified specific genes whose activities are associated with chromatin targeting to certain nuclear structure (e.g., Hsp70 transgene^39^). Recently Falk et al.^40^ also postulated the roles of molecular determinants for the global changes of chromatin spatial compartmentalization. However, our analysis on potentially important TF regulators and histone marks for cell-type specific subcompartments provides promising opportunity to further narrow down the search space for molecular determinants that modulate subcompartment variation across cell types. In addition, it is possible to develop predictive models based on SNIPER subcompartments to prioritize important sequence features, which can be validated by rewiring experiments. Overall, our work demonstrated that SNIPER has the potential to become a useful tool to offer new perspectives of 3D genome organization changes in different cell types and cellular conditions.

## Methods

### The denoising autoencoder for inferring high-coverage inter-chromosomal Hi-C contacts

We aim to recover missing contacts from sparse inter-chromosomal Hi-C contact maps by constructing a denoising autoencoder^19^ (Fig. 1b), which uses rows of the downsampled Hi-C contact probability matrix (see later section) in GM12878 as inputs, and targets the corresponding rows of the dense matrix. Here we use inter-chromosomal Hi-C contacts instead of intra-chromosomal contacts because subcompartment-specific patterns are much more visually distinct in the inter-chromosomal Hi-C matrix as compared to the intra-chromosomal Hi-C matrix, greatly facilitating subcompartment identification (see Supplementary Notes and Supplementary Fig. 21). Each row of the matrix is a vector of contact probabilities between one locus in an odd (or even) chromosome and all loci in the even (or odd) chromosomes. The denoising autoencoder in SNIPER contains a total of 9 sequential layers with ${N}_{{\mathrm{loci}}}$, 1024, 512, 256, 128, 256, 512, 1024, and ${N}_{{\mathrm{loci}}}$ neurons, respectively, where ${N}_{{\mathrm{loci}}}$ refers to the number of rows or columns in the input contact matrix. Layers with ${N}_{{\mathrm{loci}}}$ neurons are the input and output layers, the layer with 128 neurons is the latent layer, and the remaining layers are hidden layers. The autoencoder network is trained using a moderate coverage Hi-C matrix obtained by randomly removing 90% of the original GM12878 Hi-C read pairs to reflect the sparsity and coverage levels of other cell types. We input a subset of $N$ total rows into the input layer of the autoencoder. Its output layer targets corresponding rows in the high-coverage Hi-C matrix. The layers of the encoder and decoder, pertaining, respectively, to the layers before and after the latent layer of the autoencoder, contain 14–15 million parameters, approximately 10% of the $N\times {N}_{{\mathrm{loci}}}\approx 180$ million sparse inputs in the training matrix. The 128 dimension of the latent layer limits the number of parameters in the autoencoder to approximately match our downsampling ratio of 1:10, and enables the downstream classifier to accurately predict subcompartment annotations.

*Linear transformations to compute neural layer outputs*. We developed a denoising autoencoder that is comprised of linear layers with neurons whose activations are computed by:

1 $${{\bf{z}}}_{i}({{\bf{x}}}_{i})={g}_{i}({{\bf{W}}}_{i}{{\bf{x}}}_{i}+{{\bf{b}}}_{i})$$

where ${{\bf{z}}}_{i}$ is the activated output of layer $i$, ${{\bf{x}}}_{i}$ is the $n$-dimensional input to layer $i$, ${{\bf{W}}}_{i}$ is the $m\times n$-dimensional weight matrix (where $m$ is the layer’s output dimensionality) of layer $i$, ${{\bf{b}}}_{i}$ is the $m$-dimensional bias vector of layer $i$, and ${g}_{i}$ is the activation function applied element-wise to the output vector for layer $i$.

*Nonlinear activation to promote separability and consistency of autoencoder outputs*. We apply rectified linear unit (ReLU) activation^41^ to the hidden layers:

2 $$\,\text{ReLU}\,({\bf{z}})=\left\{\begin{array}{cc}{\bf{z}}&({\bf{z}} \, > \, 0)\\ 0&({\bf{z}}\le 0)\end{array}\right.$$

where all non-positive values in the output vector ${\bf{z}}$ are set to 0 to introduce sparsity. Sparse neural activation is less entangled, more linearly separable, and more efficiently propagates information throughout the network. In addition, ReLU has been shown to be suitable for naturally sparse data^42^.

Of the hidden layers, those with 1024 and 256 neurons are forwarded into 25% dropout layers to reduce overfitting^43^. The latent and output layers are activated linearly and sigmoidally, respectively, with no dropout:

3 $$\sigma ({\bf{z}})=\frac{1}{1+\exp (-{\bf{z}})}$$

where the values in the activated output $\sigma ({\bf{z}})$ are constrained between 0 and 1 as the exponential converges to 0 and 1 (as values in a layer’s output $z$ go to $-\infty$ and $+\infty$). The latent layer is linearly activated to maximize the encoding space that latent variables can occupy. The output layer is sigmoidally activated to match the range of values in the input probability matrix.

*Binary cross-entropy to optimize the autoencoder*. We use binary cross-entropy (BCE) loss to assign weights to samples whose autoencoder output values deviate significantly from corresponding target values:

4 $${\hat{\theta}}=\,\arg \,\min _{\theta}\left\{-\sum _{i=1}^{N}\left({{\bf{y}}}_{i}^{T}{\mathrm{log}}({\hat{\bf{y}}}_{i})+(1-{\bf{y}}_{i})^{T}{\mathrm{log}}\left(1-{\hat{\bf{y}}}_{i}\right)\right)\right\}$$

where the autoencoder parameters $\theta$ are optimized to minimize the cross entropies between model outputs ${\hat{{\bf{y}}}}_{i}$ and target outputs ${{\bf{y}}}_{i}$ for all training inputs $i\in \{1,\ldots ,N\}$. The autoencoder can also be optimized using mean-squared error loss with little difference in performance^44^.

For implementation, gradients of the weights and biases in the model are computed using backpropagation^45^ and weights are updated using RMSProp^46^. The autoencoder is trained for 25 epochs using a batch size of 32 and learning rate of 0.001.

The training set contains the first 7000 rows in the contact probability map, which includes genomic loci in chromosomes 1, 3, 5, and 7, occupying about 51% of all loci in odd-numbered chromosomes. The remaining rows in the contact probability map form the test set. We found that using different sets of chromosomes did not significantly affect the recovery of high-coverage Hi-C data and the annotation of subcompartments (Supplementary Fig. 1). In addition, the autoencoder is re-trained using even-numbered chromosomal regions as training inputs. This new training set includes loci in chromosomes 2, 4, 6, 8, and 10 and occupy about 52% of loci in even-numbered chromosomes. We transpose the downsampled Hi-C matrix, compute its probability maps, and follow the same training process, targeting the transposed high-coverage Hi-C probability map. The re-trained autoencoder model outputs the same Hi-C map as the initial model (Supplementary Fig. 22). The size of the input and output layers is adjusted to equal the number of contacts between each even-numbered chromosomal region and all odd-numbered regions.

### The classifier for predicting Hi-C subcompartment annotations

We developed a multilayer perceptron model with two hidden layers to classify latent representations of inter-chromosomal contacts into subcompartment assignments (Fig. 1c). The MLP network contains layers with 128, 64, 16, and 5 neurons. The 128-neuron layer pertains to the input layer, the 64-neuron and 32-neuron layers are the hidden layers, and a 5-neuron layer is the output layer (corresponding to five primary subcompartments). The network is trained using the latent representations of inter-chromosomal contacts and the corresponding GM12878 subcompartment labels from Rao et al.^5^. We then input the 128-dimensional representations of genome-wide contacts in other cell types into the trained classifier to infer their subcompartment annotations.

Sigmoid activation is applied to the input latent variables, limiting input values between 0 and 1 and mitigating bias towards high numerical inputs. We apply ReLU activation to the output of each hidden layer, which will subsequently be forwarded to 25% dropout layers. The output layer contains 5 neurons (each representing a possible subcompartment annotation) which are activated with softmax to ensure that subcompartment probabilities summed to 1:

5 $$\sigma ({z}_{c})=\frac{\exp ({z}_{c})}{{\sum }_{j=1}^{C}\exp ({z}_{j})}$$

where the exponential activation of a class $c$, $\exp ({z}_{c})$, is normalized by the sum of exponential activation across all $C$ classes, ${\sum }_{j=1}^{C}\exp ({z}_{j})$. The output likelihoods indicate the most likely annotation of a 100 kb genomic bin.

The training set is balanced (see Methods below) to ensure that each subcompartment is equally represented in the training set. Our classifier uses a balanced set of latent representations of the same loci used to train the autoencoder as inputs and targets their corresponding subcompartment annotations ${\bf{y}}$ based on high-coverage Hi-C in Rao et al.^5^. We validated the model by comparing the predicted annotations of the latent variables of the remaining loci to the Rao et al.^5^ annotations. The model is optimized using categorical cross-entropy loss between predicted and target outputs:

6 $${{\mathcal{L}}}_{{\mathrm{MLP}}}({\bf{y}},\hat{{\bf{y}}})=-\sum _{i=1}^{N}\sum _{c\in C}{{\bf{y}}}_{i}[c]{\mathrm{log}} \, {\hat{\bf{y}}}_{i}[c]$$

where $\hat{{\bf{y}}}$ is the predicted output, ${\bf{y}}$ is the target output, and $c\in C$ are the possible output classes. The loss function sums over the class-specific entropy loss ${{\bf{y}}}_{i}[c]{\mathrm{log}} \, {\hat{{\bf{y}}}}_{i}[c]$ for all classes in each training sample $i\in \{1,\ldots ,N\}$. The weights in the classifier are updated by computing gradients of the loss function with respect to the weights:

7 $$\hat{\theta }=\,\arg\, \min_{\theta }{{\mathcal{L}}}_{{\mathrm{MLP}}}({\bf{y}},\hat{{\bf{y}}})$$

where $\theta$ is the set of model weights. Each epoch’s learning rate is adjusted using RMSProp^46^. Two independent classifiers are trained to annotate regions in odd-numbered and even-numbered chromosomes.

### Converting Hi-C contact maps into Hi-C contact probabilities

We converted Hi-C contacts into contact probabilities to mitigate the effects of extreme Hi-C signals and enable neural networks to use binary cross-entropy loss. Eq. 8 is applied element-wise to an inter-chromosomal Hi-C map, returning a matrix of contact probabilities ${P}_{ij}$ constrained between 0 and 1.

8 $${P}_{ij}=\exp \left(-\frac{1}{{C}_{ij}}\right)$$

where ${C}_{ij}$ refers to the contact frequency between genomic loci $i$ and $j$. Contacts probabilities with values constrained between 0 and 1 allow the weights of a neural network to be optimized using BCE loss instead of mean-squared-error (MSE) loss and mitigate the effects of extreme outliers. High frequency chromatin contacts can disproportionately influence activation in a neural network with linear neurons, leading to incorrect chromatin subcompartment annotations and a less robust neural network. Even after log-normalization in Rao et al.^5^, SNIPER can still become skewed by the logarithms of extreme values. Furthermore, the value range of the input exceeds the $(0,1)$ range and pushes SNIPER to compute gradients derived from MSE loss. MSE loss optimizes for mean-squared distance between network outputs and targets, which can result in regression to the mean of the targets. Using BCE loss retains Hi-C contact patterns in the autoencoder output. SNIPER’s goal is to capture contact frequency patterns in order to infer subcompartment annotations, making optimization for patterns far more important than optimizing for mean-squared distance. In addition, extreme outliers in the contact matrix will have corresponding contact probabilities that converge to 0 or 1, values which will introduce much less bias into the autoencoder. While SNIPER’s inputs could also be constrained between 0 and 1 by applying a sigmoid function to the input layer activation, the input would have to be further balanced by training an additional set of weights and biases.

Probability maps were computed for cell types GM12878, K562, IMR90, HeLa, HUVEC, HMEC, HSPC, T-Cells, and HAP1. Because the Hi-C coverage of GM12878 is much higher compared to the other cell types, we simulated the sparsity of the Hi-C maps of other cell types by downsampling GM12878’s inter-chromosomal matrix by 1:10 (and in some cell types by 1:20) and computing its downsampled probability map. The downsampled probability matrix serves as the training input for the SNIPER autoencoder and GM12878’s dense matrix serves as its target output during training.

### Training set balancing

Before training the classifier, the training set was balanced so that each subcompartment was equally represented to remove bias towards specific subcompartments. We set the number of samples per subcompartment to be a number $N$ that is greater than the number of regions in the most common subcompartment in the GM12878 training set. We then define an array $B$ corresponding to the balanced training set containing $5\times N$ training samples–$N$ samples per subcompartment.

For each of the five primary subcompartments $c$, we randomly sample two latent variables $x$ and $y$ of chromatin regions that belong to subcompartment $c$. We subsequently compute $r$, a vector novel to the training set whose values lie at a random point in between the values $x$ and $y$, i.e.,

9 $$r=x+(y-x)\times \,\text{rand}\,(0,1)$$

where rand $(0,1)$ is a random variable sampled from a uniform distribution between 0 and 1. We then append $r$ to $B$ and repeat random sampling for $N-1$ iterations. $N$ random samples are then taken for each of the remaining subcompartments.

### Methods of comparing SNIPER results in different cell types

*Transition of histone marks near subcompartment boundaries*. Epigenomic marks can serve as indicators of the overall accuracy of predicted annotations, even though they are not perfectly predictive of subcompartment state. We compiled histone marks ChIP-seq fold change in genomic regions within 400 kb of subcompartment boundaries, defined as nucleotide positions where subcompartment annotations of adjacent 100 kb chromatin regions are different.

*Conserved and dynamic subcompartment annotations across multiple cell types*. In this work, we applied SNIPER to nine cell lines—GM12878, K562, IMR90, HeLa, HUVEC, HMEC, HSPC, T cells, and HAP1—to determine regions with more conserved or more dynamic subcompartment annotations across multiple cell types. We divide subcompartment annotations in thirteen conservation states based on the entropy of each 100 kb region cross cell type annotations as follows:

10 $${S}_{i}=\sum _{c=1}^{C}\left(-{p}_{i,c}{\mathrm{log}}{p}_{i,c}\right)$$

11 $${p}_{i,c}=\frac{{\sum }_{j=1}^{N}\delta ({a}_{i,j},c)}{N}$$

where ${S}_{i}$ is the total entropy of region $i$ subcompartment annotations, summed over the entropy of all $C$ subcompartments. The fraction of subcompartment $c$ at region $i$, ${p}_{i,c}$, is computed by counting the number of occurrences of subcompartment $c$ over all $N$ cell types, ${\sum }_{j=1}^{N}\delta ({a}_{i,j},c)$, and dividing by the total number of cell types $N$. $\delta ({a}_{i,j},c)=1$ if the annotation ${a}_{i,j}$ of cell type $j$ is equal to $c$ at region $i$.

Because annotations are discrete, Eqs. 10 and 11 yielded 23 possible entropy values, each corresponding to a unique distribution of annotations across cell types. Of these 23 states, 11 are associated with fewer than 5 out of 9 cell types sharing the same subcompartment annotation. The 11 states without a majority subcompartment are merged into a single non-conserved (NC) state. We sort the remaining 13 states in order of entropy, with the lowest entropy state 1 denoting the most conserved cross cell type regions, and the higher-numbered states denoting less conserved and more dynamic regions.

To represent subcompartment conservation and dynamics, we computed information content of each 100 kb region. Information content is computed similar to entropy, but normalizing subcompartment-specific fractions by a background probability within the logarithm term:

12 $${\mathrm{I}}{{\mathrm{C}}}_{i,c}=\left|\left({p}_{i,c}{\mathrm{log}}\frac{{p}_{i,c}}{{q}_{c}}\right)\right|$$

where ${\mathrm{I}}{{\mathrm{C}}}_{i,c}$ is the information content of subcompartment $c$ at region $i$, ${p}_{i,c}$ is computed in Eq. 11, and ${q}_{c}=0.2$ is the background probability of subcompartments assuming uniform subcompartment distribution. High information content corresponds to regions with more conserved annotations while low information content corresponds to more dynamic regions across cell types.

### Reporting summary

Further information on research design is available in the Nature Research Reporting Summary linked to this article.

## Supplementary information

Supplementary Information

Reporting Summary

Source Data

## Data Availability

The Hi-C data of GM12878, K562, IMR90, HeLa, HUVEC, and HMEC were obtained from Rao et al.^5^ in GEO accession GSE63525 The Hi-C data of HSPC was obtained from Joeng et al.^47^: https://s3.amazonaws.com/hicfiles/external/goodell/HSPC.hic The Hi-C data of T cells was obtained from Joeng et al.^47^: https://s3.amazonaws.com/hicfiles/external/goodell/tcell.hic The Hi-C data of HAP1 was obtained from Sanborn et al.^32^: https://hicfiles.s3.amazonaws.com/hiseq/hap1/in-situ/combined.hic We used the Juicebox tool^48^ to extract 100 kb inter-chromosomal contacts from .hic files. Note that the Hi-C data of HSPC, T Cells, and HAP1 are only accessible using Juicebox. The GM12878 .hic file was created by concatenating the read pairs text files of HIC001–HIC029 found in GEO accession GSE63525) into a single read pairs file and using the Pre function in Juicer^49^. The processed input Hi-C data used in our analyses can be found at: https://cmu.app.box.com/s/n4jh3utmitzl88264s8bzsfcjhqnhaa0/folder/86847649053/ Of the included files, GM12878_combined.hic is the high-coverage Hi-C data used for training. GM12878_combined_<ds>.hic are the downsampled GM12878 Hi-C data where <ds> specifies the downsample level. For example, 0.1 denotes a dataset with 10% of the contacts in GM12878_combined.hic. Pre-trained SNIPER models for downsampling rates 0.02, 0.03, 0.04, 0.05, 0.1, 0.2, and 0.4 can be found at: https://cmu.app.box.com/s/n4jh3utmitzl88264s8bzsfcjhqnhaa0/folder/86849512020/ All annotations in genome assemblies hg19 and hg38 can be found at: https://cmu.app.box.com/s/n4jh3utmitzl88264s8bzsfcjhqnhaa0/folder/86847603885 All datasets used in this work, including Hi-C data, histone mark ChIP-seq data, and Repli-seq data, are listed in Supplementary Table 8. All other relevant data is available upon request.

## Code availability

Source code and documentation on how to train and apply SNIPER can be found at: https://github.com/ma-compbio/SNIPER
