# Supplementary material for: Revealing Hi-C subcompartments by imputing inter-chromosomal chromatin interactions
Source: Nat Commun. 2019 Nov 7;10:5069. doi: 10.1038/s41467-019-12954-4 (PMC6838123; doi:10.1038/s41467-019-12954-4)
Supplement: Supplementary file 1 — Supplementary Information [file 41467_2019_12954_MOESM1_ESM.pdf]

# Supplementary Information

## Revealing Hi-C subcompartments by imputing inter-chromosomal chromatin interactions

Kyle Xiong and Jian Ma

# Supplementary Figures

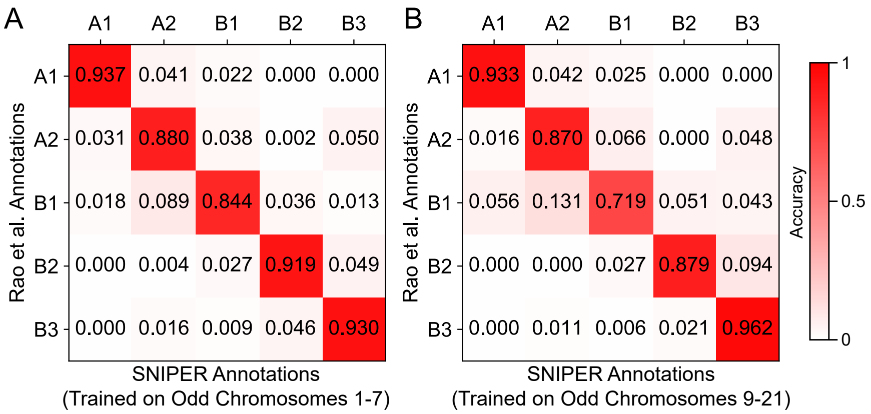


**Supplementary Figure 1:** Performance of SNIPER using different training set from GM12878. **(A)** Accuracy of SNIPER when trained using rows in chromosomes 1, 3, 5, and 7. **(B)** Accuracy of SNIPER when trained using rows in chromosomes 9, 11, 13, 15, 17, 19, and 21. Source data are available in the Source Data file.

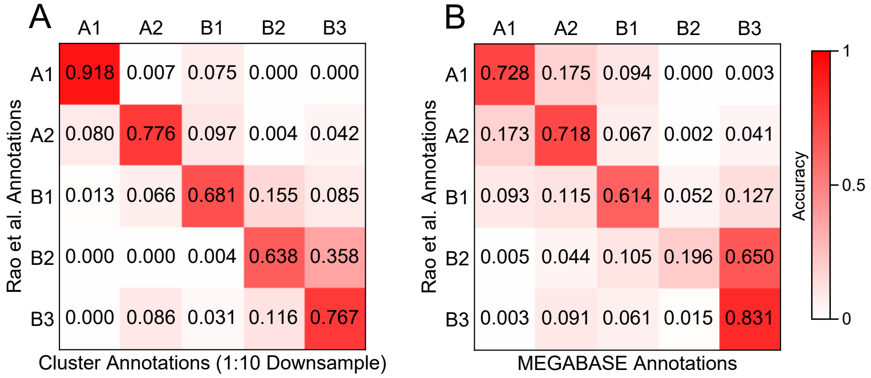


**Supplementary Figure 2:** Gaussian HMM outperforms MEGABASE when applied on GM12878 with lower coverage Hi-C. **(A)** Confusion matrix between Rao et al. (1) subcompartment annotations and annotations from a Gaussian HMM annotation based on a downsampled GM12878 (74M read pairs, i.e., 10% of the original inter-chromosomal Hi-C contacts) inter-chromosomal Hi-C matrix. **(B)** Confusion matrix between Rao et al. (1) annotations and MEGABASE annotations. Source data are available in the Source Data file.

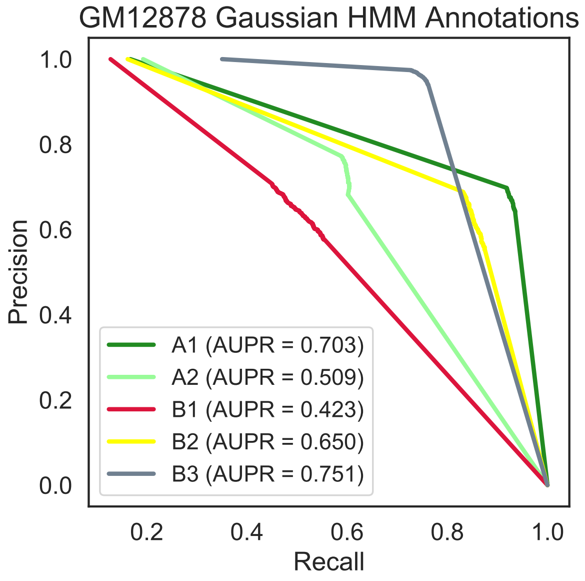


**Supplementary Figure 3:** Precision-recall curves for each subcompartment annotated by a Gaussian HMM based on the downsampled inter-chromosomal Hi-C matrix of GM12878 (10% of the original inter-chromosomal Hi-C contacts). Source data are available in the Source Data file.

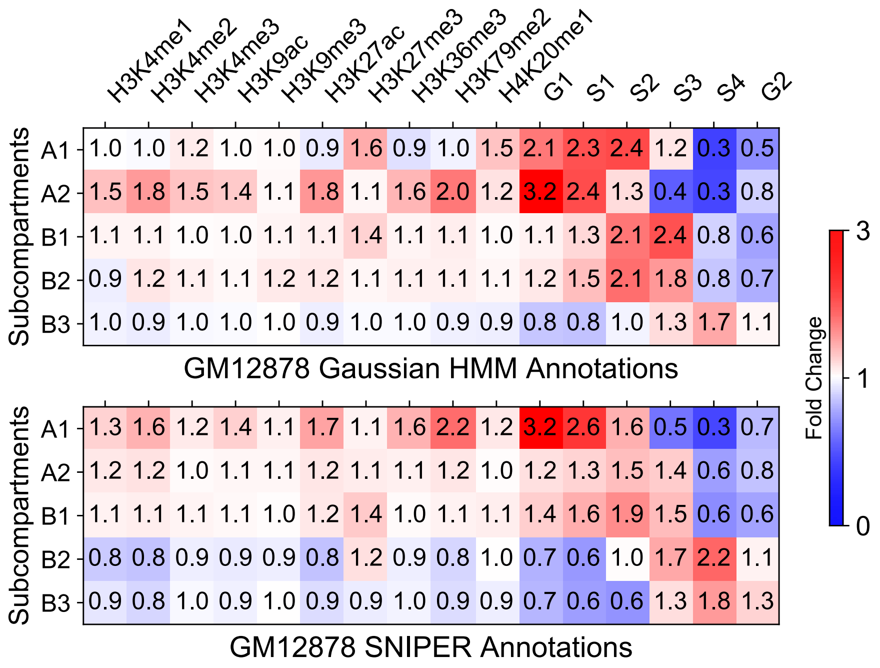


**Supplementary Figure 4:** Comparison of the enrichment of functional genomic signals in genomic regions with subcompartment annotations that are different from the reference annotation (based on the Gaussian HMM annotation from the full dataset). **(Top)** Gaussian HMM subcompartment annotation by training on the downsampled inter-chromosomal matrix of GM12878 (10% of the original inter-chromosomal Hi-C contacts). **(Bottom)** SNIPER subcompartment annotations. Note that in both panels each entry only consists of genomic regions whose subcompartment annotations are *different* from the reference annotations. Source data are available in the Source Data file.

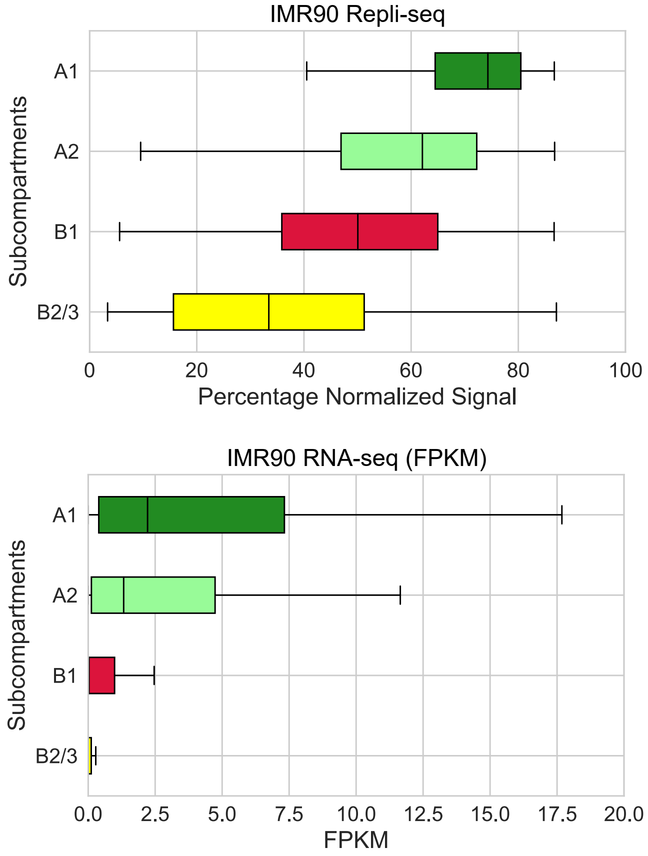


**Supplementary Figure 5:** Distribution of Repli-seq and RNA-seq FPKM for each subcompartment in IMR90. Boxes extend from the lower to upper quartiles. Center lines in each boxplot represent the median and whiskers denote 1.5 interquartile range. Source data are available in the Source Data file.

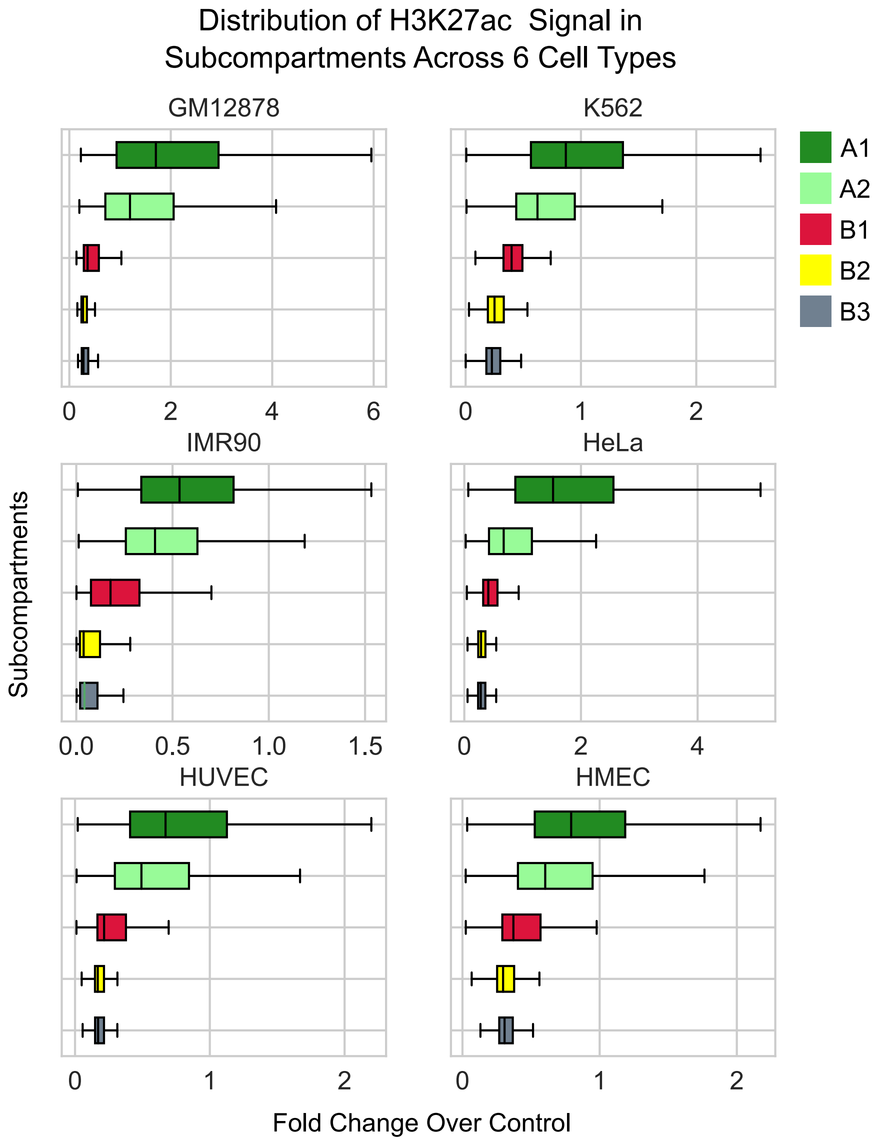


**Supplementary Figure 6:** Distribution of H3K27ac fold change over control for each subcompartment in GM12878, K562, IMR90, HeLa, HUVEC, and HMEC. Boxes extend from the lower to upper quartiles. Center lines in each boxplot represent the median and whiskers denote 1.5 interquartile range. Source data are available in the Source Data file.

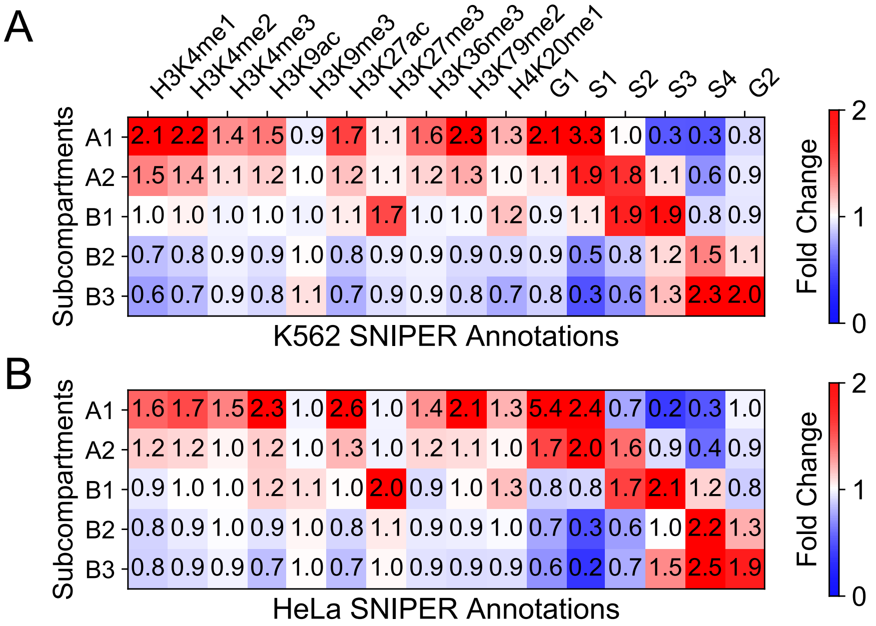


**Supplementary Figure 7:** The enrichment of functional genomic signals in SNIPER subcompartment annotations in two cancer cell lines in this work: **(A)** K562; **(B)** HeLa. Source data are available in the Source Data file.

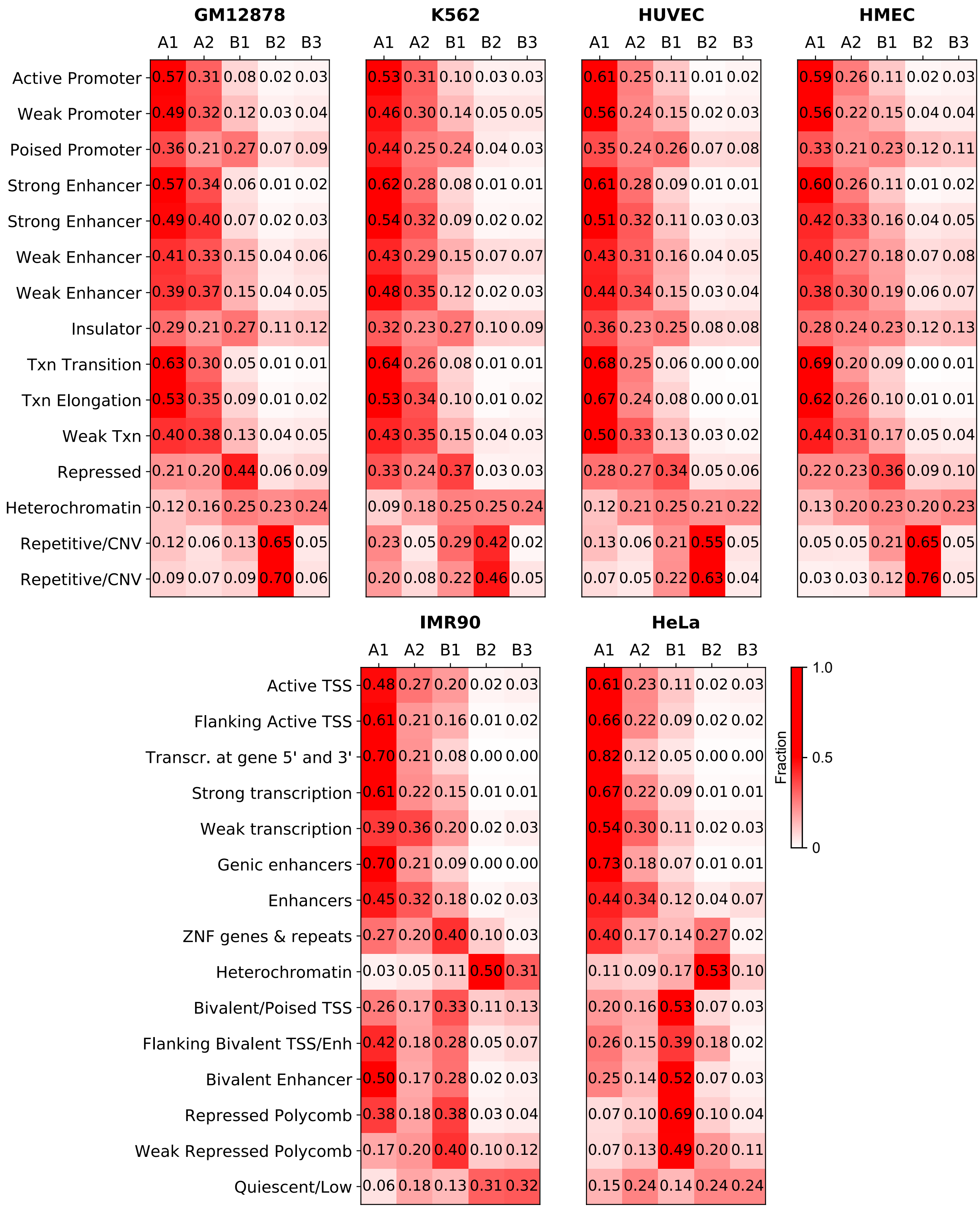


**Supplementary Figure 8:** ChromHMM state distribution in GM12878, K562, IMR90, HeLa, HUVEC, and HMEC SNIPER subcompartments. GM12878 subcompartments are the reference annotation based on Gaussian HMM while annotations in K562, IMR90, HeLa, HUVEC, and HMEC are predicted by SNIPER. Note that ChromHMM annotations for GM12878, K562, HUVEC, and HMEC have labels different from those of IMR90 and HeLa. Source data are available in the Source Data file.

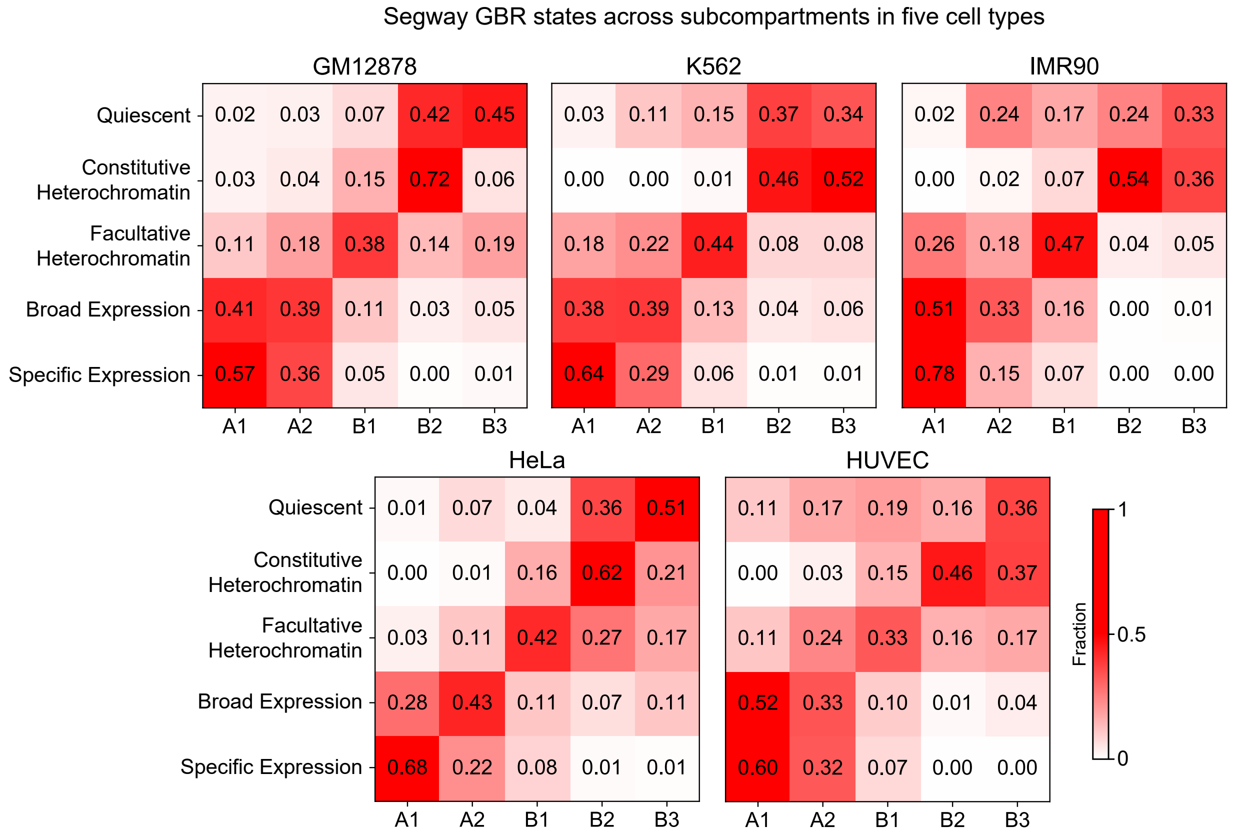


**Supplementary Figure 9:** Segway-GBR state distribution in GM12878, K562, IMR90, HeLa, and HUVEC SNIPER subcompartments. Source data are available in the Source Data file.

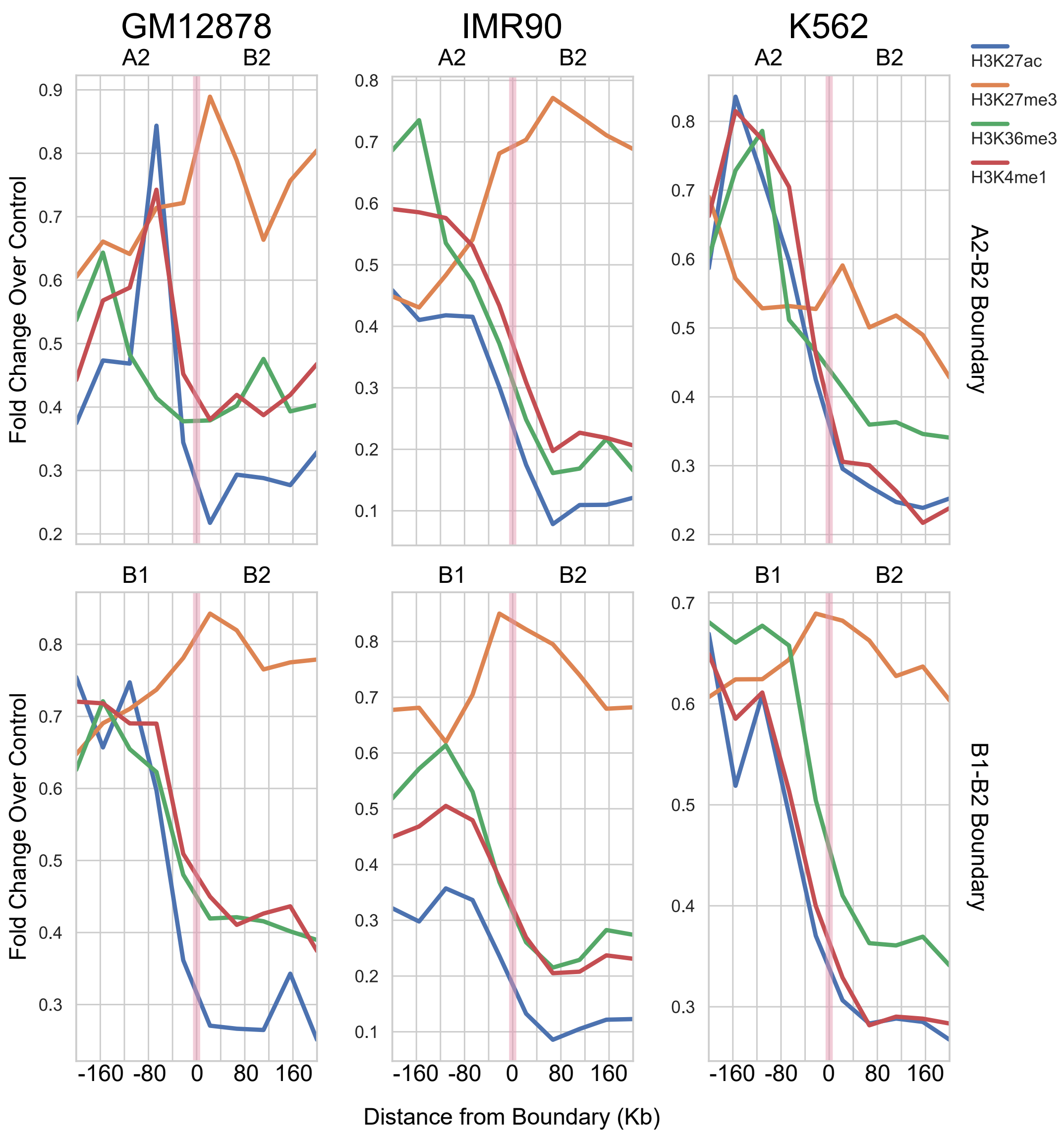


**Supplementary Figure 10:** Histone mark signals across the A2-B2 and B1-B2 boundaries in GM12878, IMR90, and K562. Source data are available in the Source Data file.

Number of Boundaries Between Subcompartments in Various Cell Types

GM12878

| | A2 | B1 | B2 | B3 |
|---|---|---|---|---|
| A1 | 329 | 591 | 9 | 1 |
| A2 | | 778 | 315 | 1042 |
| B1 | | | 306 | 49 |
| B2 | | | | 212 |

K562

| | A2 | B1 | B2 | B3 |
|---|---|---|---|---|
| A1 | 469 | 698 | 9 | 2 |
| A2 | | 734 | 198 | 697 |
| B1 | | | 558 | 144 |
| B2 | | | | 543 |

IMR90

| | A2 | B1 | B2 | B3 |
|---|---|---|---|---|
| A1 | 644 | 227 | 14 | 1 |
| A2 | | 1304 | 274 | 924 |
| B1 | | | 1087 | 242 |
| B2 | | | | 499 |

HeLa

| | A2 | B1 | B2 | B3 |
|---|---|---|---|---|
| A1 | 822 | 80 | 4 | 2 |
| A2 | | 735 | 361 | 1414 |
| B1 | | | 393 | 104 |
| B2 | | | | 578 |

HUVEC

| | A2 | B1 | B2 | B3 |
|---|---|---|---|---|
| A1 | 654 | 136 | 5 | 1 |
| A2 | | 1235 | 339 | 1380 |
| B1 | | | 637 | 190 |
| B2 | | | | 365 |

HMEC

| | A2 | B1 | B2 | B3 |
|---|---|---|---|---|
| A1 | 1174 | 170 | 8 | 0 |
| A2 | | 1305 | 307 | 1278 |
| B1 | | | 735 | 170 |
| B2 | | | | 574 |

HSPC

| | A2 | B1 | B2 | B3 |
|---|---|---|---|---|
| A1 | 171 | 451 | 8 | 0 |
| A2 | | 826 | 212 | 787 |
| B1 | | | 839 | 108 |
| B2 | | | | 769 |

T Cell

| | A2 | B1 | B2 | B3 |
|---|---|---|---|---|
| A1 | 163 | 680 | 5 | 0 |
| A2 | | 1093 | 558 | 806 |
| B1 | | | 904 | 57 |
| B2 | | | | 609 |

HAP1

| | A2 | B1 | B2 | B3 |
|---|---|---|---|---|
| A1 | 467 | 281 | 9 | 5 |
| A2 | | 764 | 311 | 1588 |
| B1 | | | 524 | 159 |
| B2 | | | | 716 |

**Supplementary Figure 11:** The number of transitions between subcompartments in GM12878, K562, IMR90, HeLa, HUVEC, HMEC, HSPC, T Cells, and HAP1. Each matrix entry shows the number of times chromatin regions transition between two subcompartments.

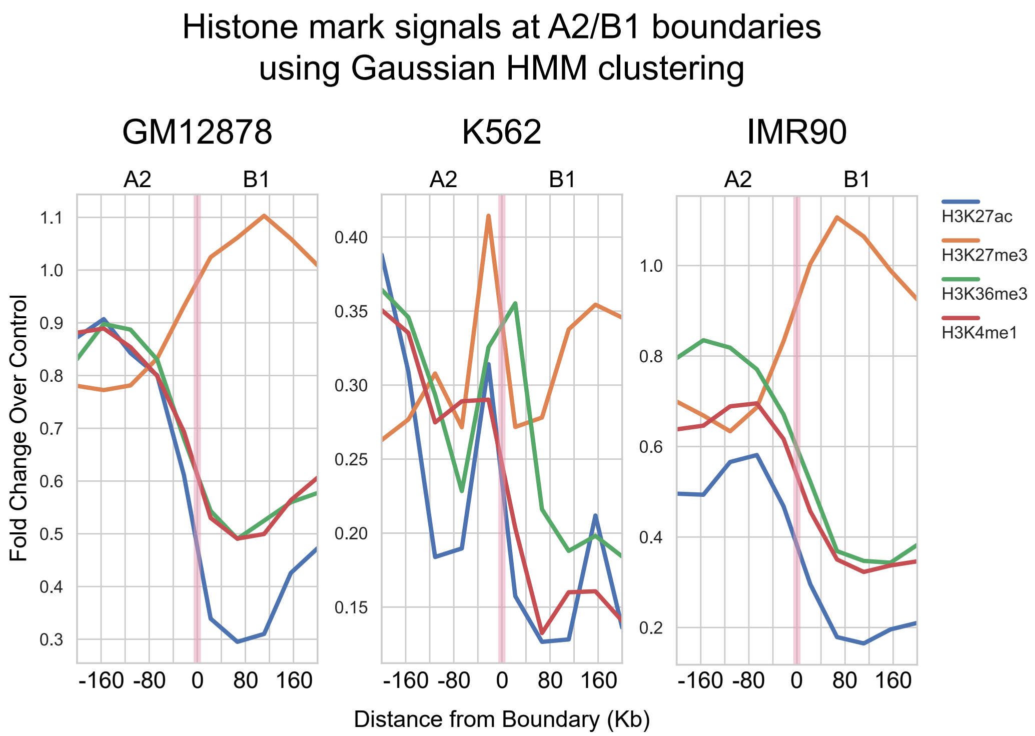


**Supplementary Figure 12:** Histone mark signals across the A2 and B1 boundary using subcompartment annotations obtained from Gaussian HMM. Source data are available in the Source Data file.

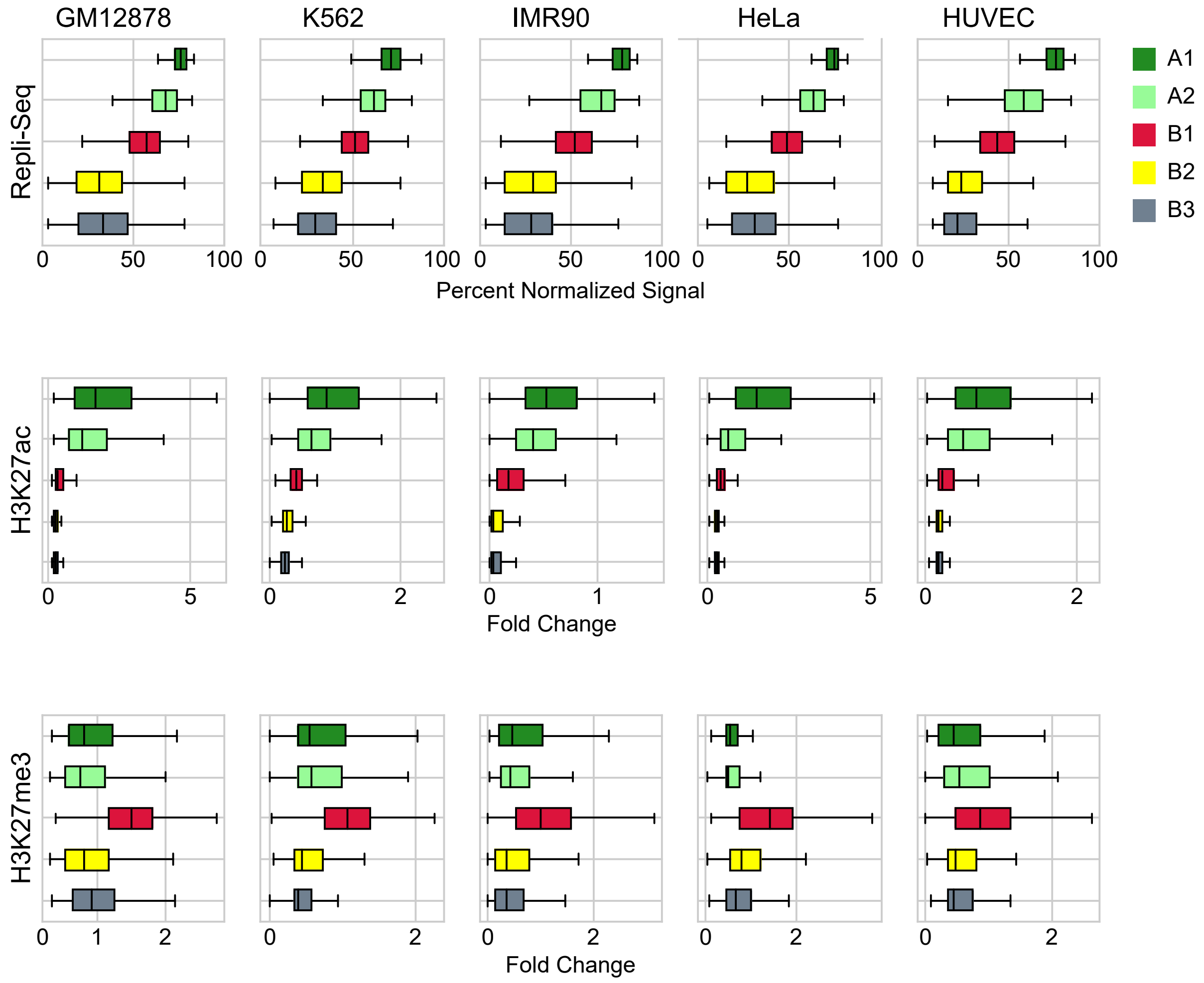


**Supplementary Figure 13:** Fold change of Repli-seq, H3K27ac ChIP-seq, and H3K27me3 ChIP-seq in SNIPER subcompartments across cell types GM12878, K562, IMR90, HeLa, and HUVEC. Boxes extend from the lower to upper quartiles. Center lines in each boxplot represent the median and whiskers denote 1.5 interquartile range. Source data are available in the Source Data file.

A

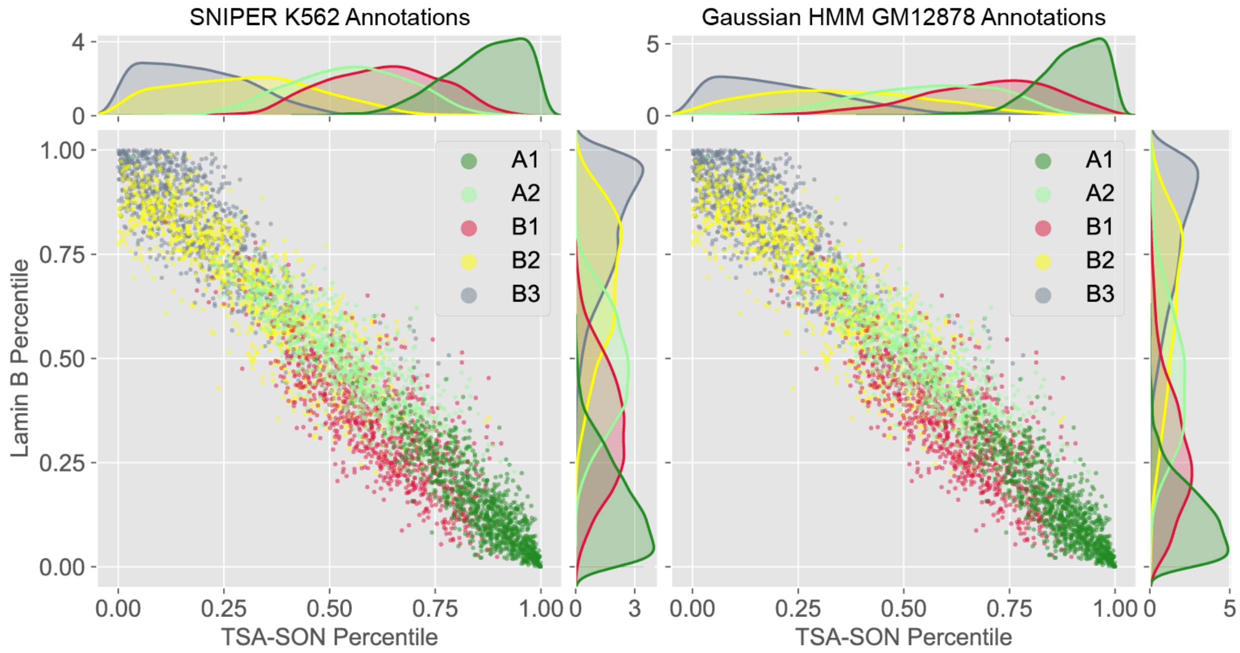


B

Variance in each subcompartment ($\times 10^{-2}$)

| | | A1 | A2 | B1 | B2 | B3 |
|---|---|---|---|---|---|---|
| **SON** | SNIPER | 0.921 | 1.932 | 1.840 | 2.871 | 1.871 |
| | Rao et al. | 0.634 | 3.072 | 2.601 | 4.007 | 2.515 |
| **Lamin B** | SNIPER | 1.078 | 1.686 | 1.970 | 2.509 | 1.761 |
| | Rao et al. | 0.714 | 2.795 | 2.574 | 4.036 | 2.308 |

**Supplementary Figure 14:** SON TSA-seq and LaminB TSA-seq signals (in K562) distribution as compared to SNIPER subcompartment annotations in K562 and the original GM12878 annotation in Rao et al. (1). **(A)** SON TSA-seq and LaminB TSA-seq signal percentile distribution in SNIPER subcompartments in K562 (left) and the original subcompartments in GM12878 (1) (right). Distribution plots along the *x* and *y* axes represent the density of SON TSA-seq and LaminB TSA-seq signal, respectively, in each subcompartment. **(B)** Variance of SON TSA-seq and LaminB SON TSA-seq signals in each subcompartment in SNIPER K562 annotations and the original subcompartments in GM12878. Source data are available in the Source Data file.

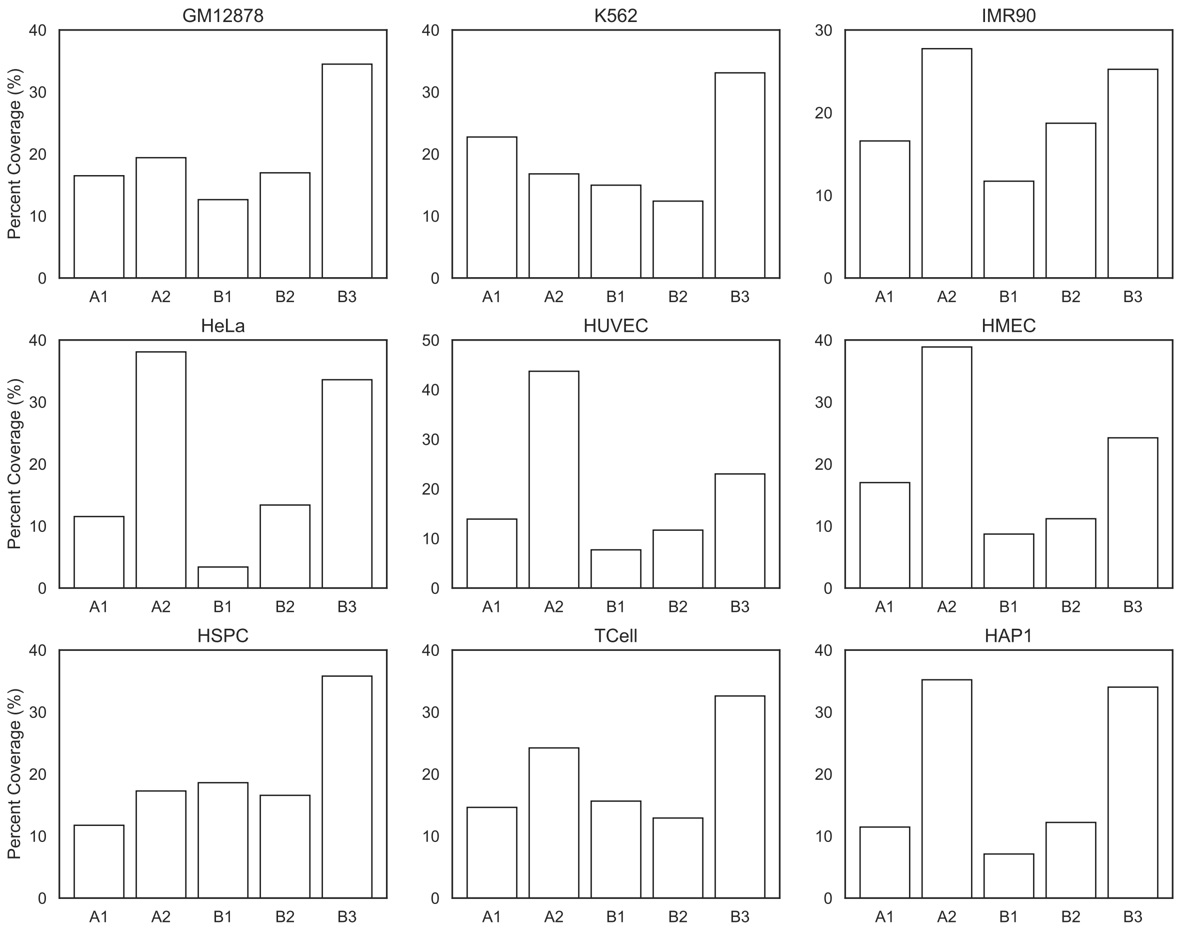


**Supplementary Figure 15:** Genome-wide distribution of each subcompartment in all 9 cell lines in this work. Source data are available in the Source Data file.

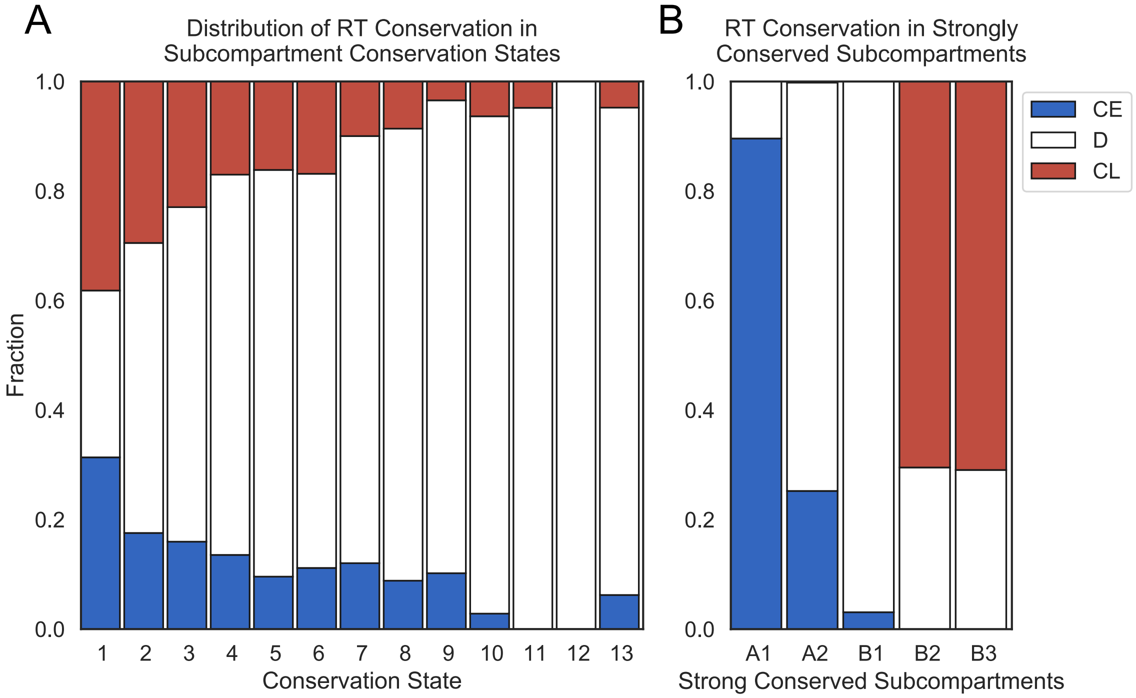


**Supplementary Figure 16:** Correlation between constitutive early (CE), developmentally regulated (D), and constitutive late (CL) replication timing (RT) states (defined based on ES cell differentiation (2)) with different subcompartment conservation levels. Fractions along the y-axes are computed by dividing the base pair overlap between RT states and conservation states by the total base pairs in each conservation state. **(A)** Correlation between RT states and subcompartment conservation states 1-13 (defined in the main text). **(B)** Correlation between RT states and subcompartments that are strongly conserved across cell types. Source data are available in the Source Data file.

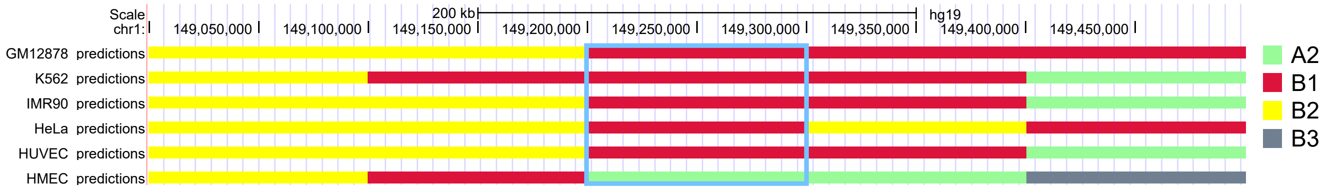


**Supplementary Figure 17:** An example of cell-type specific A2 where one cell type (HMEC) is annotated as A2 in the highlighted region and other cell types are annotated as B1. Note that this is an example of cell type of subcompartments that we used to identify potential important histone marks and transcription factor motifs among these six cell types.

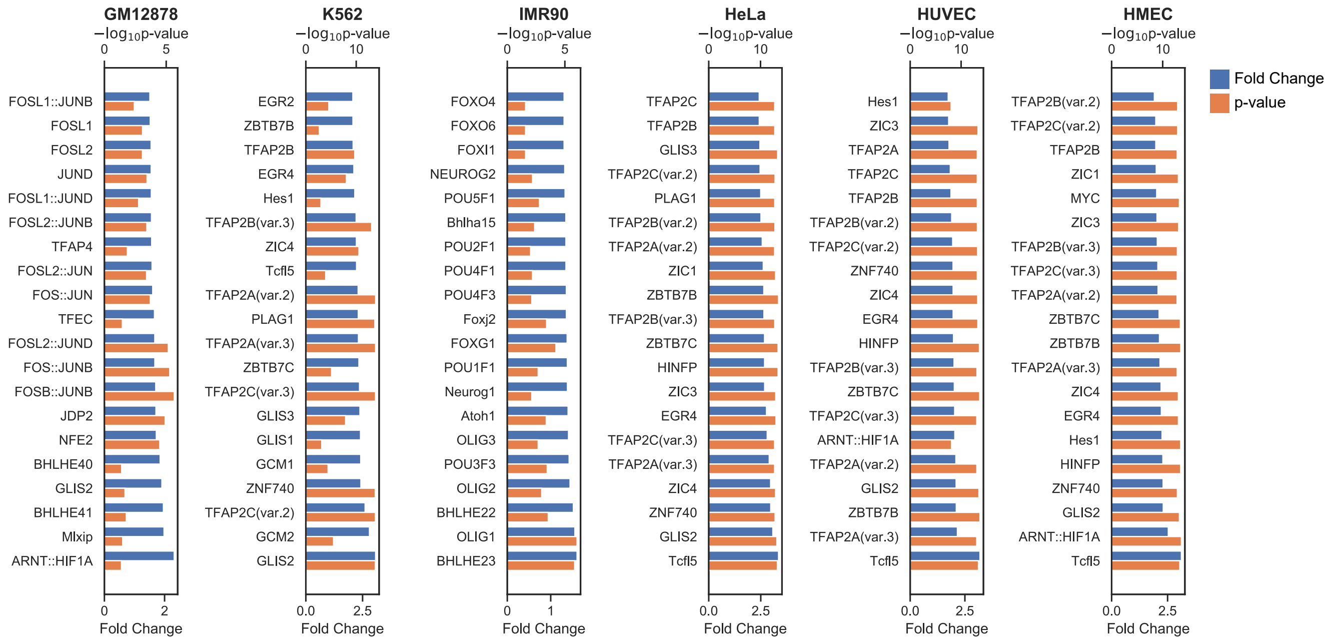


**Supplementary Figure 18:** Top 20 enriched transcription factor motifs in cell-type specific A2 subcompartments, ordered by fold-change over the background frequency of each motif (blue bars). Also shown are the $-\log_{10}$ *p*-values (binomial test) of each motif after Bonferroni correction (orange bars). Source data are available in the Source Data file.

A

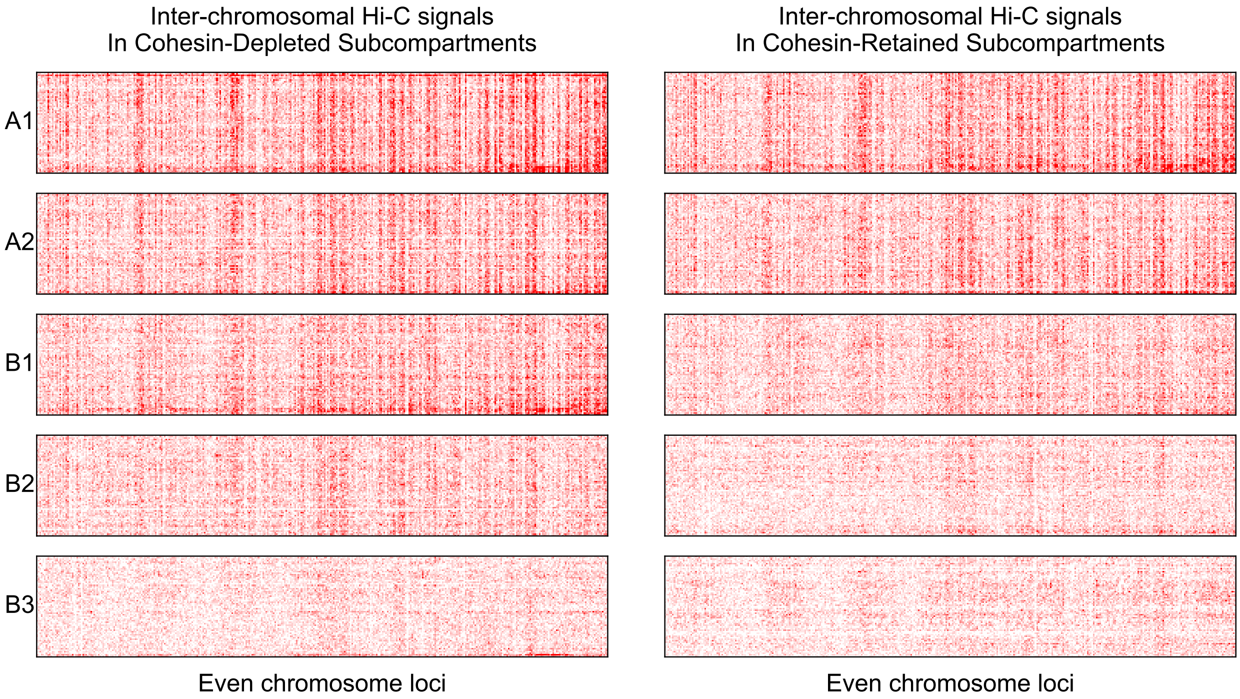


B

Normalized mean reads per interaction pair in each subcompartment

| | A1 | A2 | B1 | B2 | B3 |
|---|---|---|---|---|---|
| Cohesin-Depleted | 2.094 | 1.745 | 1.696 | 1.394 | 1.000 |
| Wild-Type | 2.054 | 1.736 | 1.466 | 1.064 | 1.000 |

**Supplementary Figure 19:** Application of SNIPER to the Hi-C dataset from cohesin-depleted and wild-type HCT116 (3). **(A)** Hi-C signals of all genomic regions in each subcompartment under cohesin-depleted and wild-type conditions (HCT116). These regions are partitioned based on what they are annotated as under each condition – cohesin-depleted or wild-type. Among these regions, A1, A2, and B1 had significantly more enhanced Hi-C signals in cohesin-depleted HCT116 whereas in wild-type HCT116, B1, B2, and B3 had very similar levels of Hi-C signals. **(B)** We computed the mean number of Hi-C read pairs per inter-chromosomal interaction in the subcompartments from both cohesin-depleted and wild-type HCT116. The mean numbers of read pairs are then divided by the mean numbers of read pairs in B3 under each condition (i.e., B3 is the control).

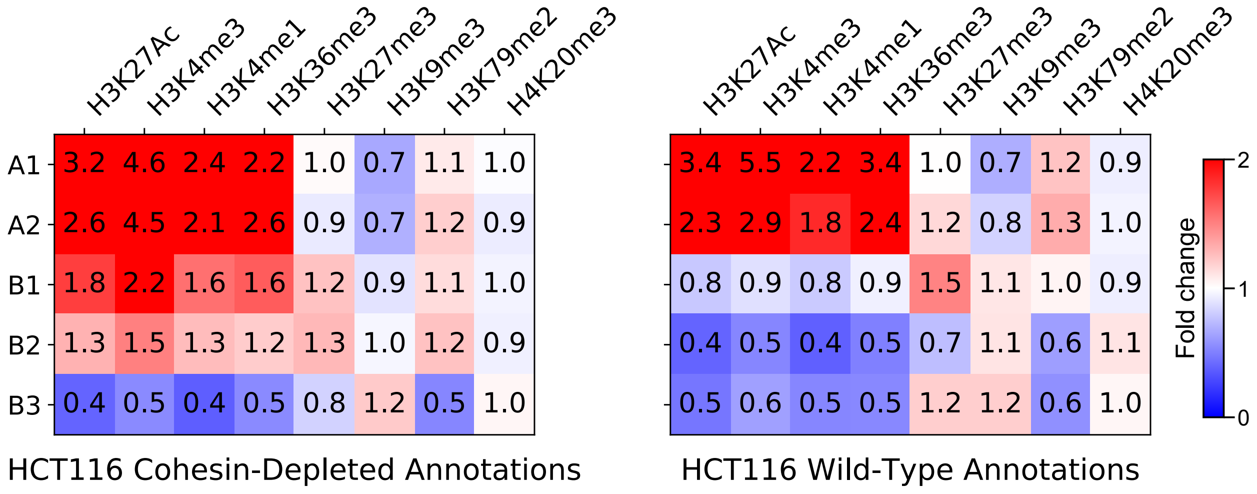


**Supplementary Figure 20:** Comparison between the histone mark enrichment of cohesin-depleted and wild-type HCT116 in 100kb chromatin regions where subcompartment annotations are different.

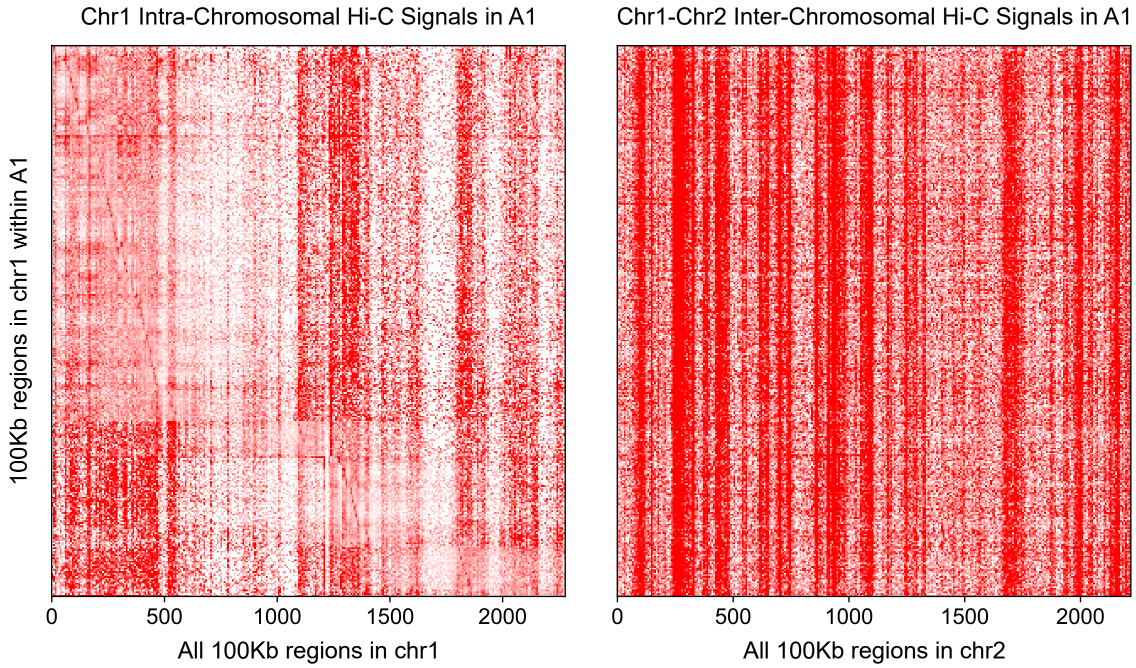


**Supplementary Figure 21:** Comparison between intra-chromosomal and inter-chromosomal Hi-C signals (in GM12878) associated with loci in the A1 subcompartment of chromosome 1. The regions in chromosome 1 that belong to A1 are along *y*-axis of both subfigures. The *x*-axis of the intra-chromosomal matrix (on the left) pertains to all regions in chromosome 1. The *x*-axis of the inter-chromosomal matrix (on the right) pertains to all regions in chromosome 2. The intra-chromosomal matrix has visibly more variance compared to the inter-chromosomal matrix between chromosome 1 along the rows and chromosome 2 along the columns.

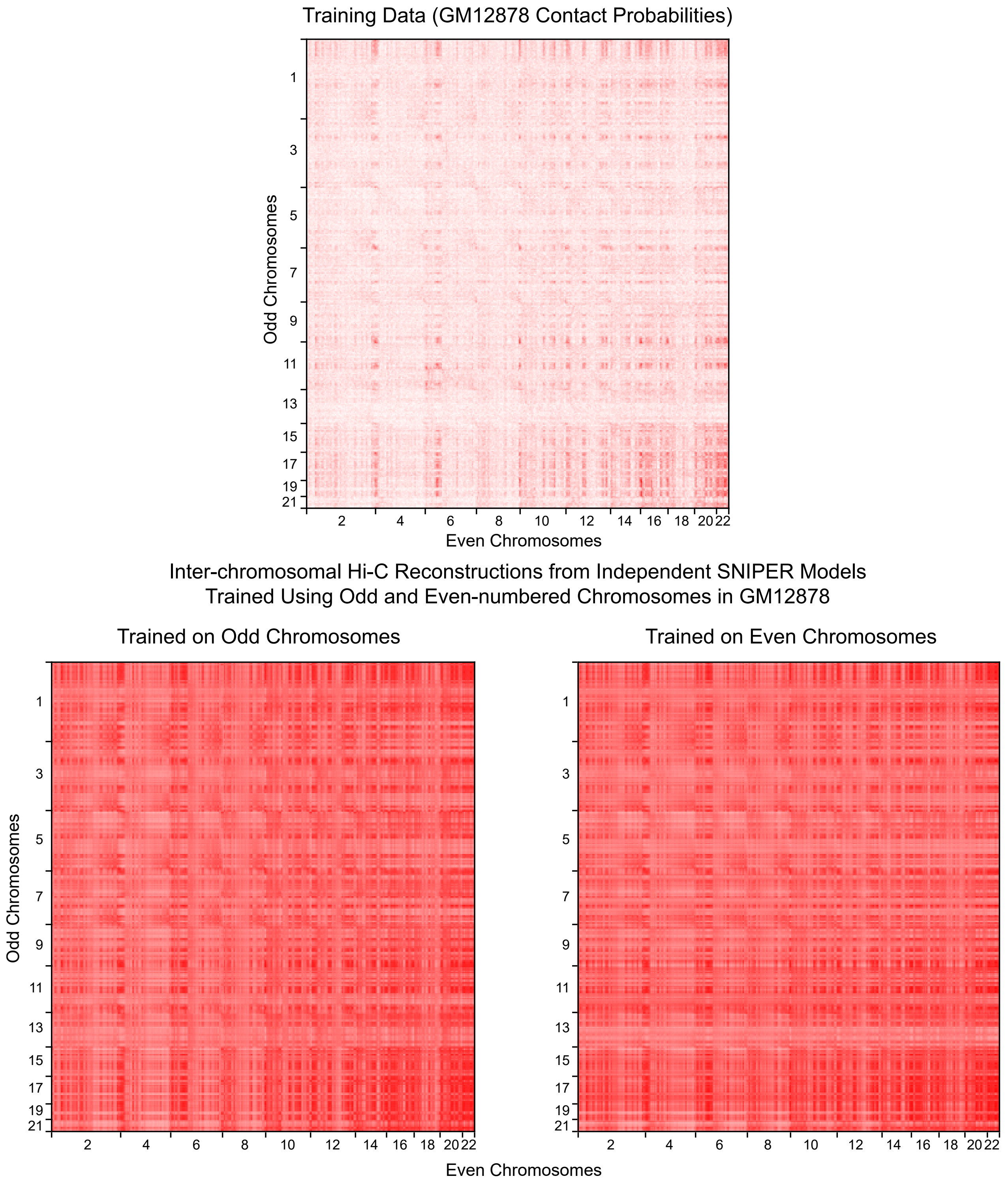


**Supplementary Figure 22:** Hi-C probabilities reconstructed from sparse training data (top) using independent SNIPER models trained using odd (bottom-left) chromosomes and even (bottom-right) chromosomes.

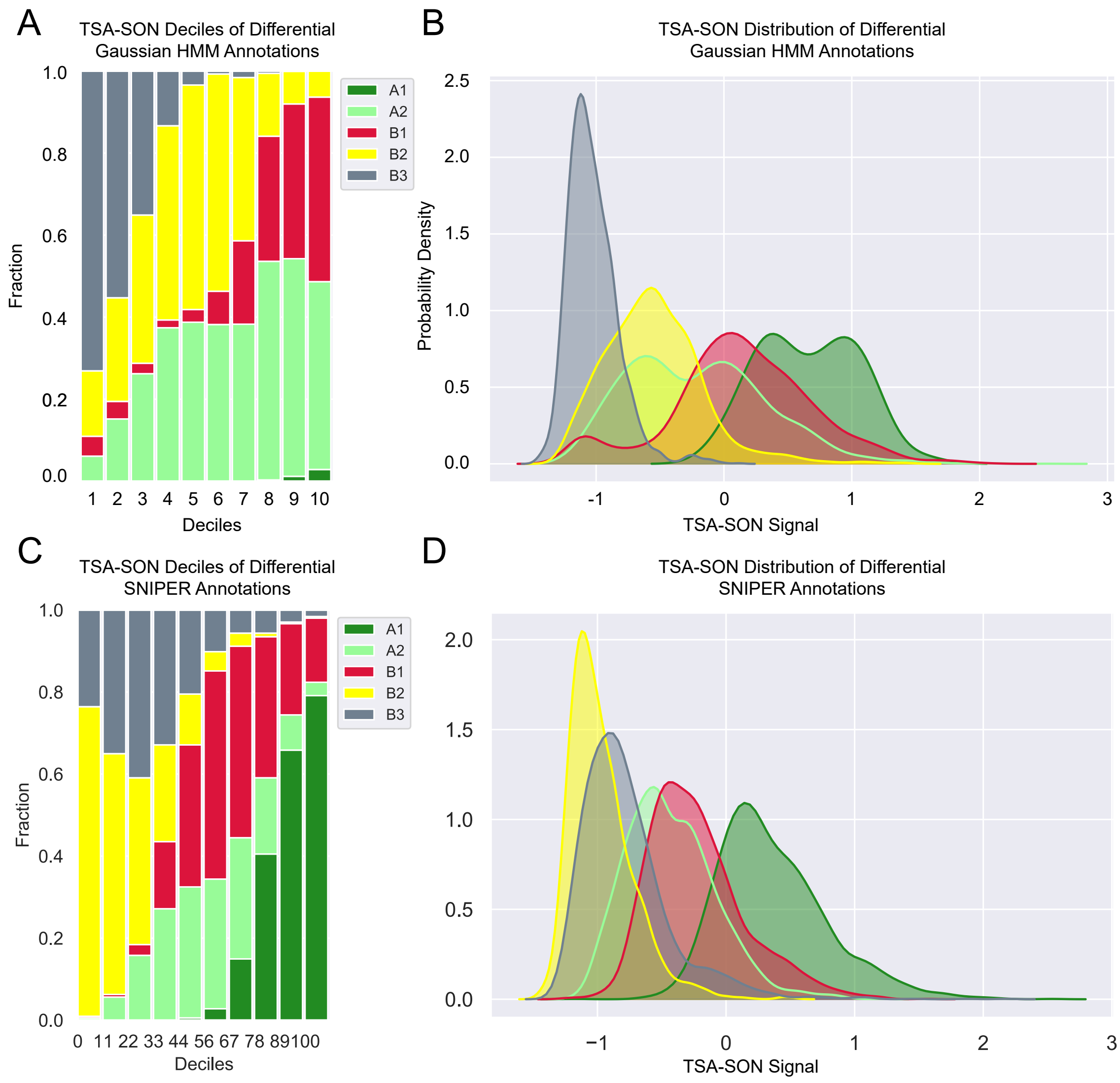


**Supplementary Figure 23:** Comparison between SON TSA-seq signals in K562 and Gaussian HMM subcompartment annotation based on K562 Hi-C data. **(A)** Gaussian HMM K562 annotations (that are different from SNIPER K562 annotations) distribution in SON TSA-seq signal deciles. **(B)** Distribution of SON TSA-seq signals in regions with Gaussian HMM annotations that are different from SNIPER annotations in K562. **(C)** SNIPER K562 annotations (that are different from Gaussian HMM K562 annotations) distribution in SON TSA-seq signal deciles. **(D)** Distribution of SON TSA-seq signals in regions with SNIPER annotations that are different from Gaussian HMM annotations in K562. Source data are available in the Source Data file.

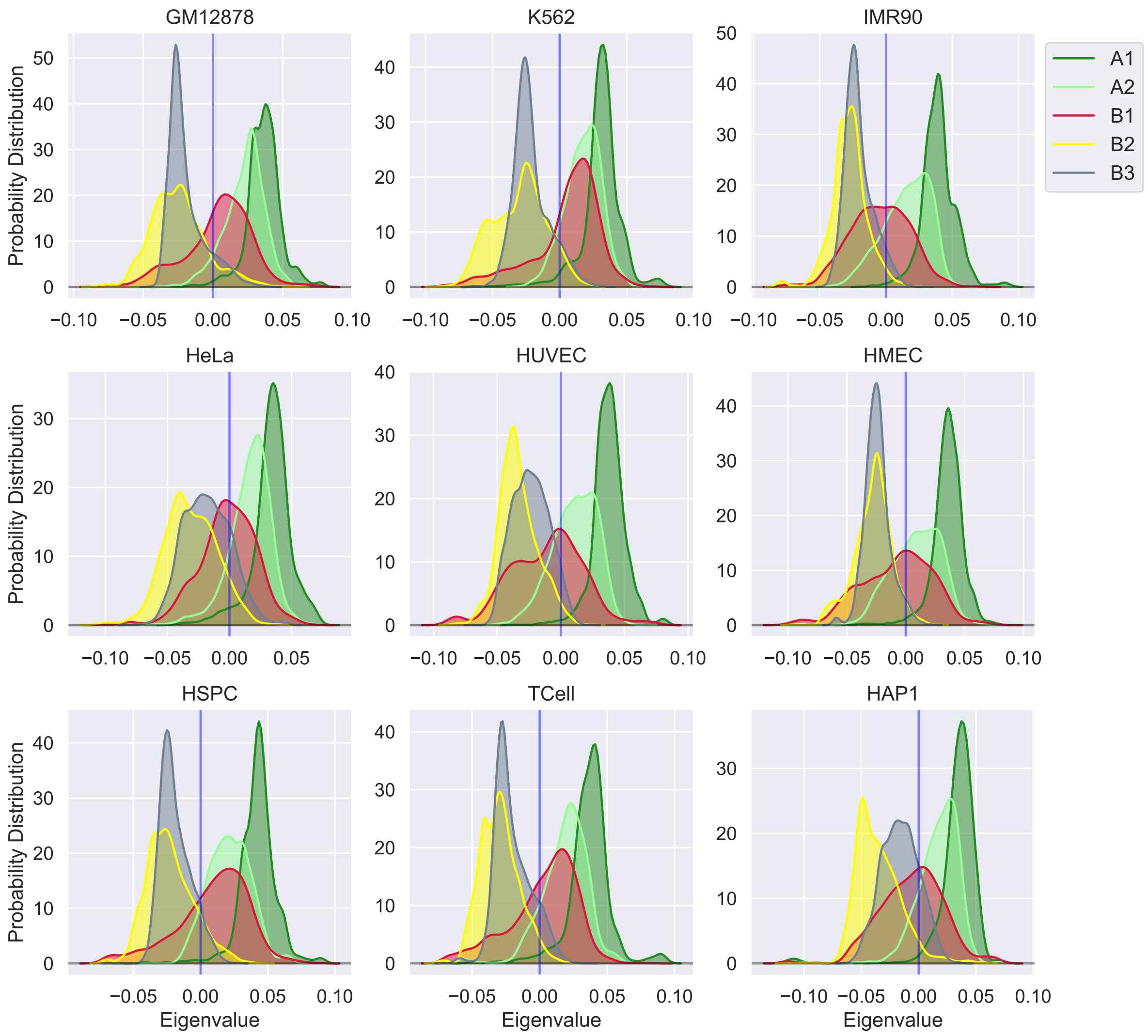


**Supplementary Figure 24:** Distribution of eigenvalues used to compute A/B compartments in each cell type. Eigenvalues are computed from the first principle component of the Pearson correlation matrix of each intra-chromosomal Hi-C matrix. Source data are available in the Source Data file.

## Supplementary Tables

A

**SNIPER Autoencoder 10-Fold Cross Validation (CE)**

| Fold | Training Loss | Test Loss |
|---|---|---|
| 1 | 0.632 | 0.636 |
| 2 | 0.630 | 0.630 |
| 3 | 0.632 | 0.633 |
| 4 | 0.631 | 0.634 |
| 5 | 0.631 | 0.633 |
| 6 | 0.632 | 0.632 |
| 7 | 0.631 | 0.633 |
| 8 | 0.630 | 0.631 |
| 9 | 0.633 | 0.634 |
| 10 | 0.634 | 0.635 |

B

**SNIPER Classifier 10-Fold Cross Validation Accuracy (%)**

| Fold | Overall | A1 | A2 | B1 | B2 | B3 |
|---|---|---|---|---|---|---|
| 1 | 91.97 | 90.50 | 94.83 | 84.80 | 90.00 | 94.93 |
| 2 | 91.02 | 94.65 | 91.89 | 87.08 | 86.89 | 91.53 |
| 3 | 92.60 | 98.25 | 91.73 | 82.69 | 88.75 | 96.71 |
| 4 | 92.44 | 95.92 | 95.19 | 87.69 | 80.42 | 94.96 |
| 5 | 91.65 | 95.30 | 89.53 | 86.17 | 82.35 | 97.24 |
| 6 | 92.36 | 97.95 | 87.45 | 87.94 | 89.68 | 95.35 |
| 7 | 92.44 | 96.75 | 88.93 | 89.25 | 89.60 | 94.83 |
| 8 | 91.81 | 96.46 | 90.78 | 84.13 | 88.98 | 94.25 |
| 9 | 92.43 | 97.15 | 90.48 | 87.17 | 86.76 | 95.08 |
| 10 | 92.75 | 97.55 | 90.73 | 84.13 | 90.84 | 96.02 |

**Supplementary Table 1:** 10-fold cross validation result of SNIPER in GM12878. **(A)** 10-fold cross validation binary cross-entropy (CE) loss of the autoencoder in SNIPER. **(B)** 10-fold cross validation accuracy for each subcompartment. Latent variable inputs are not balanced but each fold has similar accuracy to the classifier trained on the balanced training set.

| Cell Line | GM12878 | K562 | IMR90 | HeLa | HUVEC |
|---|---|---|---|---|---|
| Inter-chromosomal Reads | 741.2 | 108.1 | 94.95 | 55.41 | 71.36 |
| Cell Line | HMEC | HSPC | T Cell | HAP1 | |
| Inter-chromosomal Reads | 50.80 | 97.58 | 94.64 | 62.81 | |

**Supplementary Table 2:** Inter-chromosomal coverage (million read pairs) for various cell lines in this work. The number of reads refers to the total number of mapped read pairs between odd and even chromosomes.

| Without embedding | | | | | |
|---|---|---|---|---|---|
| | Accuracy | | | | |
| | A1 | A2 | B1 | B2 | B3 |
| MLP/SNIPER | 97.64% | 93.87% | 58.33% | 77.10% | 95.13% |
| Gaussian HMM | 63.82% | 68.18% | 57.60% | 55.22% | 94.04% |
| SVM | 87.13% | 89.71% | 42.08% | 20.47% | 99.11% |
| Random Forest | 88.67% | 81.15% | 16.95% | 25.43% | 98.46% |

| With embedding | | | | | |
|---|---|---|---|---|---|
| | Accuracy | | | | |
| | A1 | A2 | B1 | B2 | B3 |
| MLP/SNIPER | 93.86% | 89.89% | 90.11% | 87.18% | 97.16% |
| Gaussian HMM | 81.61% | 38.93% | 65.17% | 63.32% | 61.68% |
| SVM | 95.05% | 91.25% | 90.52% | 88.90% | 96.69% |
| Random Forest | 95.35% | 92.25% | 91.30% | 91.43% | 95.24% |

**Supplementary Table 3:** Performance of different classifiers/clustering methods compared to SNIPER when trained using the downsampled (10% of the original) inter-chromosomal Hi-C matrix of GM12878. **(Top)** Classifiers trained without using the SNIPER autoencoder to encode Hi-C inputs. **(Bottom)** Classifiers trained using the SNIPER autoencoder to encode Hi-C inputs.

| **GM12878** (110 genes) | | | |
|---|---|---|---|
| **Source** | **Term ID** | **Term Name** | **FDR** |
| GO:CC | GO:0019814 | immunoglobulin complex | 2.95E-03 |
| GO:CC | GO:0042571 | immunoglobulin complex, circulating | 2.95E-03 |
| GO:BP | GO:0050853 | B cell receptor signaling pathway | 3.59E-03 |
| GO:BP | GO:0050911 | detection of chemical stimulus involved in sensory perception of smell | 3.60E-03 |
| GO:BP | GO:0050864 | regulation of B cell activation | 3.60E-03 |

| **K562** (543 genes) | | | |
|---|---|---|---|
| **Source** | **Term ID** | **Term Name** | **FDR** |
| REAC | REAC:R-HSA-983231 | Factors involved in megakaryocyte development and platelet production | 1.07E-02 |
| HP | HP:0011902 | Abnormal hemoglobin | 1.63E-02 |
| WP | WP:WP3925 | Amino Acid metabolism | 1.80E-02 |
| HP | HP:0002919 | Ketonuria | 3.45E-02 |
| HP | HP:0001935 | Microcytic anemia | 3.45E-02 |

| **IMR90** (153 genes) | | | |
|---|---|---|---|
| **Source** | **Term ID** | **Term Name** | **FDR** |
| GO:BP | GO:0060379 | cardiac muscle cell myoblast differentiation | 2.81E-03 |
| GO:BP | GO:0007507 | heart development | 2.81E-03 |
| GO:BP | GO:0003256 | regulation of transcription from RNA polymerase II promoter involved in myocardial precursor cell differentiation | 2.81E-03 |
| GO:BP | GO:0060485 | mesenchyme development | 2.81E-03 |
| GO:BP | GO:0046849 | bone remodeling | 2.81E-03 |

| **HeLa** (364 genes) | | | |
|---|---|---|---|
| **Source** | **Term ID** | **Term Name** | **FDR** |
| GO:CC | GO:0005811 | lipid droplet | 9.79E-03 |
| GO:MF | GO:0004745 | retinol dehydrogenase activity | 2.19E-02 |

| **HUVEC** (210 genes) | | | |
|---|---|---|---|
| **Source** | **Term ID** | **Term Name** | **FDR** |
| HP | HP:0001658 | Myocardial infarction | 3.05E-02 |
| HP | HP:0001504 | Metacarpal osteolysis | 3.05E-02 |
| HP | HP:0001495 | Carpal osteolysis | 3.05E-02 |
| HP | HP:0001473 | Metatarsal osteolysis | 3.05E-02 |
| HP | HP:0005973 | Fructose intolerance | 3.05E-02 |

| **HMEC** (263 genes) | | | |
|---|---|---|---|
| **Source** | **Term ID** | **Term Name** | **FDR** |
| GO:CC | GO:0001533 | cornified envelope | 3.13E-23 |
| GO:BP | GO:0018149 | peptide cross-linking | 3.87E-18 |
| GO:BP | GO:0030216 | keratinocyte differentiation | 8.87E-13 |
| GO:BP | GO:0009913 | epidermal cell differentiation | 5.92E-12 |
| GO:BP | GO:0031424 | keratinization | 1.27E-11 |

| **T Cell** (202 genes) | | | |
|---|---|---|---|
| **Source** | **Term ID** | **Term Name** | **FDR** |
| GO:BP | GO:0002250 | adaptive immune response | 5.07E-12 |
| GO:BP | GO:0006955 | immune response | 1.76E-05 |
| GO:BP | GO:0002376 | immune system process | 2.29E-04 |
| GO:MF | GO:0015271 | outward rectifier potassium channel activity | 1.02E-02 |
| GO:MF | GO:0005251 | delayed rectifier potassium channel activity | 1.25E-02 |

| **Conserved** (2741 genes) | | | |
|---|---|---|---|
| **Source** | **Term ID** | **Term Name** | **P-value** |
| GO:BP | GO:0044237 | cellular metabolic process | 1.537E-17 |
| GO:BP | GO:0034641 | cellular nitrogen compound metabolic process | 2.736E-17 |
| GO:BP | GO:0008152 | metabolic process | 2.391E-14 |
| GO:BP | GO:0006807 | nitrogen compound metabolic process | 9.576E-14 |
| GO:BP | GO:0044238 | primary metabolic process | 6.133E-12 |

**Supplementary Table 4:** GO analysis results for regions in cell-type specific SNIPER A1 and A2 in GM12878, K562, IMR90, HeLa, HUVEC, HMEC, and T Cells. The bottom table shows the GO analysis of conserved subcompartments obtained from g:Profiler. FDR threshold of 0.05 is used for cell-type specific GO terms; Bonferroni corrected p-values are used for conserved GO terms. Analysis includes terms from Gene Ontology (GO), the Human Protein Atlas (HP), and Reactome (REAC). Source data are available in the Source Data file.

| GM12878 | | | |
|---|---|---|---|
| **Source** | **Term ID** | **Term Name** | **FDR** |
| GO:CC | GO:0019814 | immunoglobulin complex | 3.62E-03 |
| GO:CC | GO:0042571 | immunoglobulin complex, circulating | 3.62E-03 |
| GO:BP | GO:0050864 | regulation of B cell activation | 4.71E-03 |
| GO:BP | GO:0006956 | complement activation | 4.71E-03 |
| GO:BP | GO:0050853 | B cell receptor signaling pathway | 4.71E-03 |

| K562 | | | |
|---|---|---|---|
| **Source** | **Term ID** | **Term Name** | **FDR** |
| REAC | REAC:R-HSA-983231 | Factors involved in megakaryocyte development and platelet production | 1.93E-02 |
| HP | HP:0011902 | Abnormal hemoglobin | 3.01E-02 |

| IMR90 | | | |
|---|---|---|---|
| **Source** | **Term ID** | **Term Name** | **FDR** |
| GO:BP | GO:0036302 | atrioventricular canal development | 1.24E-02 |
| GO:BP | GO:0008284 | positive regulation of cell population proliferation | 1.24E-02 |
| GO:BP | GO:0090184 | positive regulation of kidney development | 1.24E-02 |
| GO:BP | GO:0003256 | regulation of transcription from RNA polymerase II promoter involved in myocardial precursor cell differentiation | 1.24E-02 |
| GO:BP | GO:0007507 | heart development | 1.24E-02 |

| HeLa | | | |
|---|---|---|---|
| **Source** | **Term ID** | **Term Name** | **FDR** |
| GO:CC | GO:0030914 | STAGA complex | 1.00E-02 |
| GO:CC | GO:0005811 | lipid droplet | 1.00E-02 |
| GO:CC | GO:0070461 | SAGA-type complex | 2.44E-02 |
| GO:MF | GO:0016616 | oxidoreductase activity, acting on the CH-OH group of donors, NAD or NADP as acceptor | 2.59E-02 |
| GO:MF | GO:0016614 | oxidoreductase activity, acting on CH-OH group of donors | 2.59E-02 |

| HUVEC | | | |
|---|---|---|---|
| **Source** | **Term ID** | **Term Name** | **FDR** |
| HP | HP:0001658 | Myocardial infarction | 3.05E-02 |
| HP | HP:0001504 | Metacarpal osteolysis | 3.05E-02 |
| HP | HP:0001495 | Carpal osteolysis | 3.05E-02 |
| HP | HP:0001473 | Metatarsal osteolysis | 3.05E-02 |
| HP | HP:0005973 | Fructose intolerance | 3.05E-02 |

| HMEC | | | |
|---|---|---|---|
| **Source** | **Term ID** | **Term Name** | **FDR** |
| GO:CC | GO:0001533 | cornified envelope | 6.45E-22 |
| GO:BP | GO:0018149 | peptide cross-linking | 2.84E-17 |
| GO:BP | GO:0030216 | keratinocyte differentiation | 8.94E-12 |
| GO:BP | GO:0009913 | epidermal cell differentiation | 5.87E-11 |
| GO:BP | GO:0031424 | keratinization | 8.85E-11 |

| T Cell | | | |
|---|---|---|---|
| **Source** | **Term ID** | **Term Name** | **FDR** |
| GO:BP | GO:0002250 | adaptive immune response | 3.60E-09 |
| GO:BP | GO:0006955 | immune response | 2.01E-04 |
| GO:BP | GO:0002376 | immune system process | 1.94E-03 |
| GO:MF | GO:0015271 | outward rectifier potassium channel activity | 2.63E-02 |
| GO:MF | GO:0003700 | DNA-binding transcription factor activity | 2.63E-02 |

| Conserved | | | |
|---|---|---|---|
| **Source** | **Term ID** | **Term Name** | **P-value** |
| GO:BP | GO:0044237 | cellular metabolic process | 1.709E-17 |
| GO:BP | GO:0034641 | cellular nitrogen compound metabolic process | 7.956E-17 |
| GO:BP | GO:0008152 | metabolic process | 1.949E-14 |
| GO:BP | GO:0006807 | nitrogen compound metabolic process | 2.183E-13 |
| GO:BP | GO:0044238 | primary metabolic process | 6.359E-12 |

**Supplementary Table 5:** GO analysis results in GM12878, K562, IMR90, HeLa, HUVEC, HMEC, T Cells, and conserved subcompartments obtained from g:Profiler without any subcompartment filter. Analysis includes terms from Gene Ontology (GO), the Human Protein Atlas (HP), and Reactome (REAC). Source data are available in the Source Data file.

| | | **GM12878** (71 genes) | |
|---|---|---|---|
| Source | Term ID | Term Name | FDR |
| GO:BP | GO:0009607 | response to biotic stimulus | 6.32E-06 |
| GO:BP | GO:0051707 | response to other organism | 6.32E-06 |
| GO:BP | GO:0043207 | response to external biotic stimulus | 6.32E-06 |
| GO:CC | GO:0042613 | MHC class II protein complex | 7.17E-06 |
| GO:CC | GO:0042611 | MHC protein complex | 1.03E-05 |

| | | **K562** (156 genes) | |
|---|---|---|---|
| Source | Term ID | Term Name | FDR |
| GO:MF | GO:0031720 | haptoglobin binding | 3.50E-06 |
| GO:CC | GO:0031838 | haptoglobin-hemoglobin complex | 5.54E-06 |
| GO:CC | GO:0005833 | hemoglobin complex | 5.98E-06 |
| GO:MF | GO:0005344 | oxygen carrier activity | 1.13E-05 |
| GO:MF | GO:0004601 | peroxidase activity | 3.53E-05 |

| | | **IMR90** (115 genes) | |
|---|---|---|---|
| Source | Term ID | Term Name | FDR |
| GO:CC | GO:0044456 | synapse part | 4.66E-03 |
| GO:CC | GO:0044306 | neuron projection terminus | 4.66E-03 |
| GO:CC | GO:0045202 | synapse | 6.81E-03 |
| GO:CC | GO:0005887 | integral component of plasma membrane | 6.81E-03 |
| GO:CC | GO:0098793 | presynapse | 6.81E-03 |

| | | **HeLa** (175 genes) | |
|---|---|---|---|
| Source | Term ID | Term Name | FDR |
| GO:BP | GO:0098655 | cation transmembrane transport | 1.81E-04 |
| GO:BP | GO:0006812 | cation transport | 3.77E-04 |
| GO:BP | GO:0030001 | metal ion transport | 4.93E-04 |
| GO:BP | GO:0055085 | transmembrane transport | 4.93E-04 |
| GO:BP | GO:0006811 | ion transport | 4.93E-04 |

| | | **HUVEC** (209 genes) | |
|---|---|---|---|
| Source | Term ID | Term Name | FDR |
| GO:MF | GO:0008179 | adenylate cyclase binding | 1.52E-02 |
| GO:MF | GO:0031997 | N-terminal myristoylation domain binding | 1.65E-02 |
| GO:BP | GO:0075206 | positive regulation by host of symbiont cAMP-mediated signal transduction | 2.21E-02 |
| GO:BP | GO:0075205 | modulation by host of symbiont cAMP-mediated signal transduction | 2.21E-02 |
| GO:BP | GO:0052526 | positive regulation by organism of signal transduction in other organism involved in symbiotic interaction | 2.21E-02 |

| | | **HMEC** (108 genes) | |
|---|---|---|---|
| Source | Term ID | Term Name | FDR |
| GO:BP | GO:0043588 | skin development | 3.55E-25 |
| GO:BP | GO:0031424 | keratinization | 1.68E-24 |
| GO:BP | GO:0030216 | keratinocyte differentiation | 2.89E-23 |
| GO:BP | GO:0070268 | cornification | 1.57E-22 |
| GO:BP | GO:0009913 | epidermal cell differentiation | 8.67E-22 |

| | | **T Cell** (410 genes) | |
|---|---|---|---|
| Source | Term ID | Term Name | FDR |
| GO:BP | GO:0002250 | adaptive immune response | 1.98E-51 |
| GO:BP | GO:0006955 | immune response | 7.91E-26 |
| GO:BP | GO:0002376 | immune system process | 1.04E-20 |
| GO:MF | GO:0003823 | antigen binding | 1.05E-14 |
| GO:MF | GO:0034987 | immunoglobulin receptor binding | 1.71E-14 |

| | | **Conserved A1 and A2** (2846 genes) | |
|---|---|---|---|
| Source | Term ID | Term Name | P-value |
| GO:BP | GO:0044237 | cellular metabolic process | 3.235E-15 |
| GO:BP | GO:0008152 | metabolic process | 2.353E-10 |
| GO:BP | GO:0034641 | cellular nitrogen compound metabolic process | 2.480E-10 |
| GO:BP | GO:0006807 | nitrogen compound metabolic process | 9.951E-10 |
| GO:BP | GO:0044249 | cellular biosynthetic process | 2.940E-09 |

**Supplementary Table 6:** GO terms returned by querying genes found in cell-type specific A1 and A2 annotations based on Gaussian HMM. Genes with FPKM at least two standard deviations above the mean FPKM of the same genes in other cell types are added to the GO query set. GO enrichment terms are found using g:Profiler with a FDR cutoff of 0.05. Source data are available in the Source Data file.

| Source | Term ID | Term Name | FDR |
|---|---|---|---|
| **GM12878** | | | |
| **Source** | **Term ID** | **Term Name** | **FDR** |
| REAC | REAC:R-HSA-6809371 | Formation of the cornified envelope | 3.03E-02 |
| REAC | REAC:R-HSA-6805567 | Keratinization | 3.03E-02 |
| **K562** | | | |
| **Source** | **Term ID** | **Term Name** | **FDR** |
| GO:CC | GO:0030849 | autosome | 2.14E-02 |
| GO:MF | GO:0051378 | serotonin binding | 2.21E-02 |
| GO:MF | GO:0043176 | amine binding | 2.21E-02 |
| GO:CC | GO:0001741 | XY body | 2.59E-02 |
| GO:CC | GO:0045495 | pole plasm | 2.59E-02 |
| **IMR90** | | | |
| **Source** | **Term ID** | **Term Name** | **FDR** |
| GO:BP | GO:0050906 | detection of stimulus involved in sensory perception | 5.30E-03 |
| GO:BP | GO:0050433 | regulation of catecholamine secretion | 5.30E-03 |
| GO:BP | GO:0050907 | detection of chemical stimulus involved in sensory perception | 5.30E-03 |
| GO:BP | GO:0050911 | detection of chemical stimulus involved in sensory perception of smell | 5.30E-03 |
| GO:BP | GO:0007606 | sensory perception of chemical stimulus | 5.30E-03 |
| **HeLa** | | | |
| **Source** | **Term ID** | **Term Name** | **FDR** |
| HP | HP:0012071 | Abnormal circulating acetylcarnitine concentration | 9.27E-03 |
| HP | HP:0030796 | Increased C-peptide level | 9.27E-03 |
| HP | HP:0006929 | Hypoglycemic encephalopathy | 9.27E-03 |
| HP | HP:0030781 | Increased circulating free fatty acid level | 9.27E-03 |
| HP | HP:0008180 | Mildly elevated creatine kinase | 9.27E-03 |
| **HUVEC** | | | |
| No terms found | | | |
| **HMEC** | | | |
| **Source** | **Term ID** | **Term Name** | **FDR** |
| GO:MF | GO:0005295 | neutral amino acid:sodium symporter activity | 1.68E-02 |
| GO:MF | GO:0015375 | glycine:sodium symporter activity | 1.68E-02 |
| GO:MF | GO:0046982 | protein heterodimerization activity | 1.68E-02 |
| GO:BP | GO:1903804 | glycine import across plasma membrane | 4.48E-02 |
| GO:BP | GO:0072272 | proximal/distal pattern formation involved in metanephric nephron development | 4.48E-02 |
| **T Cell** | | | |
| **Source** | **Term ID** | **Term Name** | **FDR** |
| HP | HP:0002555 | Absent pubic hair | 2.51E-02 |
| HP | HP:0002597 | Abnormality of the vasculature | 2.51E-02 |
| HP | HP:0005092 | Streaky metaphyseal sclerosis | 2.51E-02 |
| HP | HP:0005511 | Heinz body anemia | 2.51E-02 |
| HP | HP:0007803 | Monochromacy | 2.51E-02 |
| **Conserved** | | | |
| No terms found | | | |

**Supplementary Table 7:** GO analysis results in GM12878, K562, IMR90, HeLa, HUVEC, HMEC, and T cells obtained from g:Profiler in regions with cell-type specific SNIPER B1, B2, and B3 annotations. GO terms from conserved subcompartments are obtained in conserved B1, B2, and B3 regions. Analysis includes terms from Gene Ontology (GO), the Human Protein Atlas (HP), and Reactome (REAC). Source data are available in the Source Data file.

| | | | | |
|---|---|---|---|---|
| GM12878 | | | | |
| Histone Marks | Repli-seq | ChromHMM | Segway-GBR | Hi-C Data |
| ENCSR000AKH | ENCSR000CXJ | GM12878 ChromHMM | GM12878 Segway GBR | GSE63525 |
| ENCSR000AKD | ENCSR025TZP | | | |
| ENCSR000AKG | ENCSR849LEC | | | |
| ENCSR000AKC | ENCSR214GWY | | | |
| ENCSR000AKI | ENCSR257NBQ | | | |
| ENCSR000AKA | ENCSR703BOK | | | |
| ENCSR000AKE | | | | |
| ENCSR000AOX | | | | |
| ENCSR000AKF | | | | |
| ENCSR000AOW | | | | |
| **K562** | | | | |
| Histone Marks | Repli-seq | ChromHMM | Segway-GBR | Hi-C Data |
| ENCSR000AKX | ENCSR000CXE | K562 ChromHMM | K562 Segway GBR | GSE63525_K562_combined.hic |
| ENCSR000AKQ | ENCSR958IUL | | | |
| ENCSR000AKR | ENCSR338BMC | | | |
| ENCSR000AKU | ENCSR589KVH | | | |
| ENCSR000AKT | ENCSR802MDS | | | |
| ENCSR000AKS | ENCSR810JIM | | | |
| ENCSR000AKV | | | | |
| ENCSR000APE | | | | |
| ENCSR000APD | | | | |
| ENCSR000AKP | | | | |
| **IMR90** | | | | |
| Histone Marks | Repli-seq | ChromHMM | Segway-GBR | Hi-C Data |
| ENCSR002YRE | ENCSR000CXF | ENCFF147PPH | IMR90 Segway GBR | GSE63525_IMR90_combined.hic |
| ENCSR804SRO | ENCSR554HRW | | | |
| ENCSR431UUY | ENCSR903LKJ | | | |
| ENCSR826QYR | ENCSR874RPM | | | |
| ENCSR087PFU | ENCSR816UHB | | | |
| ENCSR055ZZY | ENCSR333KJW | | | |
| ENCSR219MYH | | | | |
| ENCSR437ORF | | | | |
| ENCSR713QLX | | | | |
| ENCSR672XZZ | | | | |
| **HeLa** | | | | |
| Histone Marks | Repli-seq | ChromHMM | Segway-GBR | Hi-C Data |
| ENCSR000APW | ENCSR000CXH | ENCFF654HNG | HeLa Segway GBR | GSE63525 |
| ENCSR000AOH | ENCSR811MTR | | | |
| ENCSR340WQU | ENCSR789SBW | | | |
| ENCSR000AOG | ENCSR628LNL | | | |
| ENCSR000AQO | ENCSR441OPI | | | |
| ENCSR000AOE | ENCSR098AZD | | | |
| ENCSR000DTY | | | | |
| ENCSR000DTZ | | | | |
| ENCSR000AOC | | | | |
| ENCSR000AOI | | | | |
| **HUVEC** | | | | |
| Histone Marks | Repli-seq | ChromHMM | Segway-GBR | Hi-C Data |
| ENCSR000AKK | | HUVEC ChromHMM | HUVEC Segway GBR | GSE63525_HUVEC_combined.hic |
| ENCSR000ALD | | | | |
| ENCSR000ALB | | | | |
| ENCSR000ALF | | | | |
| ENCSR000ALC | N/A | | | |
| ENCSR000AKN | | | | |
| ENCSR000AKM | | | | |
| ENCSR000AKL | | | | |
| ENCSR000ATB | | | | |
| ENCSR000ASD | | | | |
| **HMEC** | | | | |
| Histone Marks | Repli-seq | ChromHMM | Segway-GBR | Hi-C Data |
| ENCSR000ALY | | HMEC ChromHMM | | GSE63525_HMEC_combined.hic |
| ENCSR000AMM | | | | |
| ENCSR016JWS | | | | |
| ENCSR000ARG | | | | |
| ENCSR000ASB | N/A | | N/A | |
| ENCSR000AMK | | | | |
| ENCSR000AMJ | | | | |
| ENCSR000ALZ | | | | |
| ENCSR000ALX | | | | |
| ENCSR000ALW | | | | |

**Supplementary Table 8:** Sources of the histone mark ChIP-seq (ENCODE), Repli-seq (ENCODE), Hi-C data (GEO Accession GSE65325), ChromHMM annotation (ENCODE), and Segway GBR annotation (ENCODE) used in this work.

| | A1 | A2 | B1 | B2 | B3 |
|---|---|---|---|---|---|
| Intra-chromosomal chr1 | 0.8641 | 0.7095 | 0.7910 | 0.9014 | 0.8870 |
| Inter-chromosomal chr1 | 0.2833 | 0.3214 | 0.3762 | 0.4046 | 0.4557 |
| CV(Intra)/CV(Inter) | 3.051 | 2.207 | 2.103 | 2.228 | 1.947 |

**Supplementary Table 9:** Coefficient of variation (CV) in each subcompartment for intra-chromosomal and inter-chromosomal matrices of chromosome 1. Higher values of CV indicate more variability in the Hi-C data associated with each subcompartment. The ratio of intra-chromosomal CV to inter-chromosomal CV also shows that intra-chromosomal Hi-C is much more variable than inter-chromosomal Hi-C.

# Supplementary Notes

## Gaussian HMM applied on lower coverage Hi-C is less consistent than SNIPER

To contrast with results in Figure 2B, we computed an AUPR of 0.559 for cluster predictions using Gaussian HMM (see Supplementary Figure 3) trained on a GM12878 inter-chromosomal Hi-C matrix with 10% of the original Hi-C contacts. We note that SNIPER's AUPR of 0.977 when trained on the same dataset (see Figure 2) is significantly higher. To compare the newly trained Gaussian HMM results to the reference annotations, each cluster in the new Gaussian HMM is compared to the reference annotations and assigned the subcompartment it is most similar to. Note that the reference annotations are obtained by applying a Gaussian HMM to the GM12878 inter-chromosomal Hi-C matrix with the full dataset.

To contrast with results in Figure 2C, we computed functional genomic signals in clusters predicted by Gaussian HMM. The results are largely similar between SNIPER annotations and Gaussian HMM annotations. To better highlight the performance difference between SNIPER and Gaussian HMM, we show that regions predicted by SNIPER which are different from the reference annotations retain epigenomic features that are still characteristic of the correct predicted subcompartments (see Supplementary Figure 4). However, Gaussian HMM annotations that are different do not demonstrate the same overall enrichment of the correct subcompartments. This result demonstrates the robustness of SNIPER annotations.

To contrast with TSA-seq results shown in Figure 3C, we fit a Gaussian HMM using the K562 inter-chromosomal matrix and matched its cluster annotations to subcompartment annotations. Using the Gaussian HMM annotations that are different from the SNIPER annotations, we computed SON TSA-seq signal deciles, as shown in Supplementary Figure 23A. When comparing the SON TSA-seq signal deciles to SNIPER K562 annotations that are different from Gaussian HMM K562 annotations, it becomes clear that the results from SNIPER are much more consistent with the TSA-seq data (Supplementary Figure 23C).

## Comparison between SNIPER subcompartments and A/B compartments

We sought to evaluate the subcompartment assignments across cell types by comparing the SNIPER subcompartment annotations with the corresponding A/B compartment assignments (Supplementary Figure 24). For each chromosome in each cell type, A/B compartments (in 100kb resolution) are computed based on the first principal component of the Pearson correlation in intra-chromosomal Hi-C matrix using the approach in Lieberman-Aiden et al. (4). As expected, in all cell types, the A1 and A2 subcompartments largely correlated with the A compartment positive eigenvalues, while the B2 and B3 subcompartments mostly correspond to the B compartment negative eigenvalues.

## Comparison between SNIPER subcompartments and ChromHMM and Segway-GBR

We compared SNIPER subcompartment predictions in K562, IMR90, HeLa, HUVEC, and HMEC with chromatin states annotated by ChromHMM (5) (Supplementary Figure 8). All ChromHMM annotations were obtained from the ENCODE project. Note that there are some differences in terms of ChromHMM states across cell lines. We also include the reference subcompartment annotations in GM12878. We computed the number of overlapping base pairs between each ChromHMM state and each subcompartment in these cell lines. We divided the overlap between each ChromHMM state and subcompartment by the number of base pairs in the subcompartment to normalize the enrichment of ChromHMM states in

each subcompartment. The enrichment of each ChromHMM state across all subcompartments (each row for every cell line in Supplementary Figure 8) was then normalized to sum to 1. We found that the majority of enhancer and promoter states are more enriched in A1 and A2 subcompartments. As expected, a much smaller fraction of ChromHMM promoters and enhancers is present in B1, B2, and B3. The general trend of the ChromHMM state distributions in different subcompartments is similar across cell types with some variation. The results are largely consistent with the pattern in GM12878 based on the original Gaussian HMM subcompartment calls, which also agree with the general distribution patterns of active/repressive histone marks. However, we do observe some variation across cell types. One caveat of this analysis is that the resolution of ChromHMM states (less than a few kb) and the resolution of subcompartments (100kb) are drastically different. This analysis points to future work to develop hierarchical and integrative annotation that combines nuclear compartmentalization and chromatin states.

We then compared subcompartment annotations in GM12878, K562, IMR90, HeLa, and HUVEC with chromatin states annotated by Segway-GBR (6) with larger domain sizes (see Supplementary Figure 9). We again normalized the distribution of Segway-GBR states in each subcompartment by the size of the subcompartment. We found that the majority of broad and specific expression states are enriched in A1 and A2 subcompartments. The facultative heterochromatin state has more presence in the B1 subcompartment. Constitutive heterochromatin and quiescent states are also mainly found in the B2 and B3 subcompartments. Furthermore, the general trend of Segway-GBR state distributions in different subcompartments is similar across cell types.

### Additional comparison between SNIPER conservation patterns and replication timing across cell types

We compared the conservation from SNIPER subcompartment annotation across cell types with the constitutive and developmental replication timing (RT) patterns discovered during ES cell differentiation (2), including constitutive early (CE), constitutive late (CL), and developmentally regulated (D) RT domains. Of all RT states, 16.17% are CE, 21.05% are CL, and 62.78% are D. For genomic regions in strongly conserved subcompartments, the RT of most regions are either CE or CL (Supplementary Figure 16A). When looking just at the strongly conserved state, we show that almost all regions in strongly conserved A1 have CE RT and the majority of regions in strongly conserved B2 and B3 have CL RT. In addition, regions in strongly conserved A2 and B1 highly correspond to developmentally regulated RT regions.

### SNIPER is applied to analyze subcompartments in cohesin depleted HCT116

We used the data from Rao et al. (3) where Hi-C data from both cohesin depleted HCT116 and also the wild type are available. We did not apply the SNIPER model trained on GM12878 to HCT116 because we found that the inter-chromosomal Hi-C matrices between these two cell types are quite different. To measure the similarity between inter-chromosomal matrices, we employed a metric described in the Supplementary Methods.

We first fit a Gaussian HMM on the wild-type HCT116 interchromosomal Hi-C contact matrix (which has coverage with 420 million read pairs) to call subcompartments. We then trained a SNIPER model using a downsampled wild-type HCT116 Hi-C data with 50 million inter-chromosomal read pairs as input. We applied this SNIPER model to cohesin-depleted HCT116 Hi-C data. We identified subcompartment annotations in cohesin-depleted HCT116 with 87.92% similarity to the wild-type HCT116 subcompartments, which is consistent with the observation in Rao et al. (3) that compartmentalization remains

largely stable after cohesin depletion. However, with the subcompartment annotations from SNIPER, we were able to observe more refined subcompartment level changes between two conditions. Notably, we found that among the subcompartment annotations which *differ* between cohesin-depleted and wild-type cells, the cohesin-depleted one has more enhanced genomic and epigenomic signals at the subcompartment level as compared to the wild-type. Specifically, in these regions, inter-chromosomal Hi-C signals in each cohesin-depleted subcompartment is more enhanced than signals in wild-type subcompartments (Supplementary Figure 19), in agreement with the finding in Rao et al. (3) that loss of cohesin correlates with stronger overall genome compartmentalization. Also, to further evaluate the subcompartment calls in these two different cellular conditions, we compared with histone mark signals from respective condition (Supplementary Figure 20). The overall consistent patterns further support the SNIPER subcompartment calls in both conditions. This analysis demonstrates the capability of SNIPER for analyzing more detailed compartmentalization changes in different cellular conditions.

### Additional GO analysis of subcompartments in different cell types

We compared the GO analysis results of cell-type specific subcompartments based on SNIPER annotations and Gaussian HMM annotations. We again compiled cell-type specific annotations across these cell types and collected all significantly expressed genes which belong to any region with cell-type specific A1 and A2 annotations. In Supplementary Table 6, GO terms in K562 using annotations from Gaussian HMM include haptoglobin and hemoglobin-related terms, but do not include the disease terms shown in Supp. Table 4 which point to abnormalities due to leukemia. GM12878 is a B cell but GM12878-specific A1 and A2 GO terms based on Gaussian HMM do not include any B cell-related functions, whereas GM12878-specific A1 and A2 GO terms based on SNIPER annotation include “B cell receptor signaling pathway” and “regulation of B cell activation” which are absent from Gaussian HMM GO analysis result. Furthermore, GO terms from IMR90, HeLa, and HUVEC based on Gaussian HMM do not appear to include any cell-type specific functions. Overall, these results highlight the advantage of the subcompartments identified from SNIPER as compared to the subcompartments from Gaussian HMM.

If the subcompartment filter is removed and GO analysis includes genes from all subcompartments, terms such as microcytic anemia in K562 and cardiac muscle differentiation in IMR90 are absent from the GO enrichment results (Supplementary Table 5). Additionally, the terms that still appear have higher FDR. Overall, by including genes in all regions with cell-type specific subcompartments, the GO input set is larger but a higher proportion of the input set is not associated with cell-type specific functions previously found (when the input only includes genes in cell-type specific A1 and A2 regions).

We also performed GO enrichment analysis on cell-type specific B1, B2, and B3 regions (Supplementary Table 7). We combined all genes in cell-type specific B1, B2, and B3 regions because up-regulated genes in cell-type specific B subcompartments are frequently too few to return significant GO terms. In Supplementary Table 7, up-regulated genes in B subcompartments are not associated with cell-type specific functions, particularly in GM12878, K562, HMEC, and T cells. While the B subcompartment mostly consists of repressed genes, we found very few down-regulated genes in cell-type specific B1, B2, or B3 and virtually no cell-type specific GO terms. This may suggest that there could be much smaller number of factors that modulate B compartment globally or such regulation is more locus specific. The recent work from Falk et al. (7) also alluded to this open question.

## B4 subcompartments are primarily labeled as B1 by SNIPER

Since the original B4 annotations from Rao et al. (1) (which only occupy 0.408% of the genome) are not considered in this work, we ask what annotations SNIPER would assign to the original B4 regions. Overall, 85.45% of B4 regions are reassigned to B1 (in SNIPER), with additional 13.64% reassigned to B2 and 0.91% (one 100kb bin) assigned to A2. This suggests that vast majority of B4 regions are assigned to subcompartments in B compartments as expected, demonstrating the robustness of the SNIPER model.

## Inter-chromosomal Hi-C maps are more appropriate for subcompartment identification

As compared to inter-chromosomal Hi-C contact maps, chromosome-specific patterns found in intra-chromosomal Hi-C contact maps can confound genome-wide subcompartment identification. For example, in Supplementary Figure 21, subcompartment-specific patterns are much more visually distinct in the inter-chromosomal Hi-C matrix as compared to the intra-chromosomal Hi-C matrix. Inter-chromosomal Hi-C can better facilitate the subcompartment identification. In the intra-chromosomal matrix in Supplementary Figure 21, Hi-C interactions occur specifically in chromosome 1 but are not necessarily correlated with subcompartment specific inter-chromosomal Hi-C patterns. To further quantify the variability of intra-chromosomal and inter-chromosomal Hi-C matrices, we measured the coefficient of variation (CV), the ratio of standard deviation to mean, for each subcompartment in the intra-chromosomal and inter-chromosomal matrices of chromosome 1. Here inter-chromosomal chromosome 1 refers to contacts between regions on chromosome 1 and loci in all even-numbered chromosomes. We converted all Hi-C reads in intra-chromosomal chromosome 1 and inter-chromosomal chromosome 1 into contact probabilities. Rows in both matrices are then divided by subcompartments predicted by SNIPER in GM12878. In Supplementary Table 9, the average CV in each subcompartment across all columns in the intra-chromosomal Hi-C matrix is significantly higher than the CV in subcompartments in the inter-chromosomal matrix, indicating more variability in intra-chromosomal Hi-C contacts. This analysis confirms that inter-chromosomal Hi-C contact map is more appropriate for subcompartment identification.

## Supplementary Methods

### Methods for GO analysis of subcompartments across cell types

*Determining significantly expressed genes in cell-type specific subcompartments.* Genomic regions in the A1 and A2 subcompartments have increased gene expression in general, but they do not necessarily have high gene expression, characterized by their overall but not universally high RNA-seq signals. We compiled genes in all cell-type specific A1 and A2 subcompartments for each cell type, including GM12878, K562, IMR90, HeLa, HUVEC, HMEC, and T cells, which are the cell types with available RNA-seq data. We then filtered genes based on the z-score of their FPKM. Genes with FPKM z-score $\geq 2$ in the cell type with the cell-type specific annotation were included in the cell type GO query set. We used a background of all annotated human genes as defined in Ensembl V95. We also tried to find genes that are repressed relative to other cell types in cell-type specific B1, B2, and B3 subcompartments. We set an FPKM z-score threshold of $\leq$ -2. However, we were unable to find a significant amount of cell-type specific repressed genes.

*Determining genes in conserved subcompartments.* We compiled genes in genomic regions with the same subcompartment annotations across cell types. For genes in each conserved A1 or A2, we computed the z-score of FPKM across all cell types and added the gene to the conserved GO query set if the z-score is greater than -2 and less than 2 across all cell types to make sure that none of the genes in the GO query set for conserved subcompartment is significantly overexpressed or repressed relative to the other cell types.

### Comment on the autoencoder and the MLP classifier in SNIPER

In SNIPER, we use an autoencoder based on a neural network because this architecture has been proven to have denoising effects and effectiveness in reconstructing its input data (8). Furthermore, by training a downstream classifier on the embedding output of the encoder, we were able to achieve much greater accuracy (Supplementary Table 3, top) compared to inputting an entire row or column of the downsampled inter-chromosomal matrix into the same classifier. This result demonstrates the importance of autoencoder embedding used in our work.

When deciding which classifier to use, we compared the performance of multiple classifiers based on performance, ease of training, and extensibility. In Supplementary Table 3 (bottom), the multi-layer perceptron (MLP), SVM, and Random Forest classifiers all perform similarly when trained on autoencoder embeddings of the downsampled GM12878 inter-chromosomal Hi-C matrix. Each classifier is also easy to train, requiring no more than a few minutes to complete training. However, we eventually chose to use the MLP classifier in this work because a neural network architecture may be more extensible for future development of SNIPER. During its training process, the gradients back-propagated from the MLP network can assist in the training of the autoencoder network. The autoencoder can in principle utilize subcompartment-specific gradients to compute updates to its weight matrices and produce even better reconstructions of high-coverage Hi-C matrices. Subcompartment-specific gradients would not be available for other classifiers even though their performance is comparable to a MLP. We leave this as a future effort to improve the model.

## Predicting cell-type specific subcompartments using histone marks

Among cell types GM12878, K562, IMR90, HeLa, HUVEC, and HMEC, we searched for genomic regions where cross cell type subcompartment changes are more subtle, such as changes from B2 to B3, to identify important histone marks associated with cell-type specific subcompartment changes. Specifically, for each of these regions, we added the histone marks associated with the cell type annotated as B3 to the positive data set. The shared histone marks among all of the above cell types include H3K27ac, H3K27me3, H3K36me3, H3K4me1, H3K4me2, H3K4me3, H3K9ac, H3K9me3, H3K79me2, and H4K20m1. Each region in the positive data set is associated with the label 1. To construct a negative data set, we searched for regions where one cell type is annotated as B2 and other cell types are B3. For each of these regions, histone marks associated with the cell type annotated as B2 are added to the negative data set and are associated with the label 0.

We then combined the positive and negative datasets and balanced them by including an equal number of positive and negative samples. We trained a Random Forest (RF) classifier by randomly shuffling the combined data set and used half of the samples and their corresponding labels as the training set. The remaining half of the data set was used for testing. Training was repeated for 10 times, each with a different randomization of training and testing. The entire RF training procedure was then repeated for cell-type specific A1 and A2 and cell-type specific A2 and B1. The classifier was trained with 100 trees as more than 100 did not significantly improve prediction performance. All other parameters are the defaults specified by the RandomForestClassifier module in scikit-learn (9).

## Determining enriched TF motifs in cell-type specific subcompartment regions

Using FIMO (10) and the 573 JASPAR motifs, we searched for TF motifs in 150bp long DNase-seq peak regions in GM12878, K562, IMR90, HeLa, HUVEC, and HMEC. For each motif and in each cell type, we computed the background frequency of each motif by counting the number of occurrences of each motif in all DNase-seq peaks (genome-wide) and divided this number by the total number of DNase-seq peaks. We then calculated the motif frequency for cell-type specific subcompartments. As a proof-of-principle, we focused on regions in cell-type specific A2 subcompartments where one cell type is annotated as A2 and other cell types are annotated as any other subcompartment. For every cell type, we quantified the frequency of a motif by dividing the number of occurrences of the motif in A2 regions specific to the cell type by the total number of DNase-seq peaks present in these cell-type specific A2 regions. Fold change is then computed by dividing the frequency of the motif in cell-type specific A2 regions by its background frequency in the cell type. Motif enrichment $p$-values are calculated by binomial test.

## Determining similarity between inter-chromosomal Hi-C datasets

To determine whether the SNIPER model trained on GM12878 can be applied to the inter-chromosomal data of other cell types, we applied the following approach. After training a SNIPER model on GM12878, we applied the encoder model (in autoencoder) to the inter-chromosomal Hi-C matrix of both GM12878 and another cell type (for example, HCT116 as we did in this work). We then computed the first principle components of both embedded inter-chromosomal matrices and calculated the cosine similarity between the principle components, i.e.,

$$\text{sim} = \frac{\mathbf{pc}_1 \cdot \mathbf{pc}_2}{\|\mathbf{pc}_1\| \|\mathbf{pc}_2\|} \tag{1}$$

where $\mathbf{pc}_1$ and $\mathbf{pc}_2$ are the first principle component of the embedded inter-chromosomal Hi-C matrices in each cell type, respectively.

We found that the GM12878 encoded Hi-C matrix has between 0.89 and 0.90 similarity to other cell types used in this work except HCT116, i.e., K562, IMR90, HeLa, HUVEC, HMEC, HSPC, T cell, and HAP1. However, the GM12878 encoded Hi-C matrix only has 0.71 similarity to the one from HCT116. We deemed that HCT116 is too different from GM12878 and concluded that we could not reliably call subcompartments in HCT116 using a SNIPER model trained on GM12878. Therefore, when we applied SNIPER to HCT116 dataset to analyze the impact on subcompartments after cohesin depletion based on the data from Rao et al. (3), we trained a SNIPER model based on the wild-type HCT116 Hi-C data.

## Supplementary References


[1] Rao, S. S. *et al.* A 3d map of the human genome at kilobase resolution reveals principles of chromatin looping. *Cell* **159**, 1665–1680 (2014).

[2] Dileep, V. *et al.* Topologically associating domains and their long-range contacts are established during early g1 coincident with the establishment of the replication-timing program. *Genome Research* **25**, 1104–1113 (2015).

[3] Rao, S. S. *et al.* Cohesin loss eliminates all loop domains. *Cell* **171**, 305–320 (2017).

[4] Lieberman-Aiden, E. *et al.* Comprehensive mapping of long-range interactions reveals folding principles of the human genome. *Science* **326**, 289–293 (2009).

[5] Ernst, J. & Kellis, M. Chromhmm: automating chromatin-state discovery and characterization. *Nature Methods* **9**, 215 (2012).

[6] Libbrecht, M. W. *et al.* Joint annotation of chromatin state and chromatin conformation reveals relationships among domain types and identifies domains of cell-type-specific expression. *Genome research* **25**, 544–557 (2015).

[7] Falk, M. *et al.* Heterochromatin drives compartmentalization of inverted and conventional nuclei. *Nature* **570**, 395–399 (2019).

[8] Vincent, P., Larochelle, H., Bengio, Y. & Manzagol, P.-A. Extracting and composing robust features with denoising autoencoders. In *Proceedings of the 25th International Conference on Machine learning*, 1096–1103 (ACM, 2008).

[9] Pedregosa, F. *et al.* Scikit-learn: Machine learning in Python. *Journal of Machine Learning Research* **12**, 2825–2830 (2011).

[10] Grant, C. E., Bailey, T. L. & Noble, W. S. Fimo: scanning for occurrences of a given motif. *Bioinformatics* **27**, 1017–1018 (2011).
