# Supplementary material for: Revealing Hi-C subcompartments by imputing inter-chromosomal chromatin interactions
Source: Nat Commun. 2019 Nov 7;10:5069. doi: 10.1038/s41467-019-12954-4 (PMC6838123; doi:10.1038/s41467-019-12954-4)
Supplement: Supplementary file 2 — Reporting Summary [file 41467_2019_12954_MOESM2_ESM.pdf]

natureresearch

Corresponding author(s): Jian Ma
Last updated by author(s): Sep 22, 2019

Reporting Summary

Nature Research wishes to improve the reproducibility of the work that we publish. This form provides structure for consistency and transparency in reporting. For further information on Nature Research policies, see Authors & Referees and the Editorial Policy Checklist.

Statistics

For all statistical analyses, confirm that the following items are present in the figure legend, table legend, main text, or Methods section.

n/a	Confirmed	
☒	☐	The exact sample size (*n*) for each experimental group/condition, given as a discrete number and unit of measurement
☐	☒	A statement on whether measurements were taken from distinct samples or whether the same sample was measured repeatedly
☒	☐	The statistical test(s) used AND whether they are one- or two-sided<br>*Only common tests should be described solely by name; describe more complex techniques in the Methods section.*
☒	☐	A description of all covariates tested
☒	☐	A description of any assumptions or corrections, such as tests of normality and adjustment for multiple comparisons
☒	☐	A full description of the statistical parameters including central tendency (e.g. means) or other basic estimates (e.g. regression coefficient) AND variation (e.g. standard deviation) or associated estimates of uncertainty (e.g. confidence intervals)
☒	☐	For null hypothesis testing, the test statistic (e.g. *F*, *t*, *r*) with confidence intervals, effect sizes, degrees of freedom and *P* value noted<br>*Give P values as exact values whenever suitable.*
☒	☐	For Bayesian analysis, information on the choice of priors and Markov chain Monte Carlo settings
☒	☐	For hierarchical and complex designs, identification of the appropriate level for tests and full reporting of outcomes
☒	☐	Estimates of effect sizes (e.g. Cohen's *d*, Pearson's *r*), indicating how they were calculated

Our web collection on statistics for biologists contains articles on many of the points above.

Software and code

Policy information about availability of computer code

Data collection	Juicer Tools (1.7.6)
Data analysis	Numpy (1.15.4), Scipy (1.11.0), pyBigWig (0.3.12), matplotlib (2.2.2)

For manuscripts utilizing custom algorithms or software that are central to the research but not yet described in published literature, software must be made available to editors/reviewers. We strongly encourage code deposition in a community repository (e.g. GitHub). See the Nature Research guidelines for submitting code & software for further information.

Data

Policy information about availability of data

All manuscripts must include a data availability statement. This statement should provide the following information, where applicable:

- Accession codes, unique identifiers, or web links for publicly available datasets
- A list of figures that have associated raw data
- A description of any restrictions on data availability

Hi-C data of GM12878, K562, IMR90, HeLa, HUVEC, and HMEC were obtained from GSE63525.
Hi-C data of HSPC was obtained from https://s3.amazonaws.com/hicfiles/external/goodell/HSPC.hic.
Hi-C data of T Cells was obtained from https://s3.amazonaws.com/hicfiles/external/goodell/tcell.hic.
Hi-C data of HAP1 was obtained from https://hicfiles.s3.amazonaws.com/hiseq/hap1/in-situ/combined.hic

ChIP-Seq data was acquired from the following ENCODE data links:
https://www.encodeproject.org/files/ENCFF662QFK/@@download/ENCFF662QFK.bigWig
https://www.encodeproject.org/files/ENCFF157QTL/@@download/ENCFF157QTL.bigWig
https://www.encodeproject.org/files/ENCFF682WPF/@@download/ENCFF682WPF.bigWig
https://www.encodeproject.org/files/ENCFF828CQV/@@download/ENCFF828CQV.bigWig

https://www.encodeproject.org/files/ENCFF674XOM/@@download/ENCFF674XOM.bigWig
https://www.encodeproject.org/files/ENCFF674QZB/@@download/ENCFF674QZB.bigWig
https://www.encodeproject.org/files/ENCFF465KNK/@@download/ENCFF465KNK.bigWig
https://www.encodeproject.org/files/ENCFF846KNU/@@download/ENCFF846KNU.bigWig
https://www.encodeproject.org/files/ENCFF180LKW/@@download/ENCFF180LKW.bigWig
https://www.encodeproject.org/files/ENCFF440GZA/@@download/ENCFF440GZA.bigWig
https://www.encodeproject.org/files/ENCFF167NBF/@@download/ENCFF167NBF.bigWig
https://www.encodeproject.org/files/ENCFF552FEL/@@download/ENCFF552FEL.bigWig
https://www.encodeproject.org/files/ENCFF540UFX/@@download/ENCFF540UFX.bigWig
https://www.encodeproject.org/files/ENCFF396JIR/@@download/ENCFF396JIR.bigWig

https://www.encodeproject.org/files/ENCFF010PHG/@@download/ENCFF010PHG.bigWig
https://www.encodeproject.org/files/ENCFF840LLW/@@download/ENCFF840LLW.bigWig
https://www.encodeproject.org/files/ENCFF445UCR/@@download/ENCFF445UCR.bigWig
https://www.encodeproject.org/files/ENCFF658JMW/@@download/ENCFF658JMW.bigWig
https://www.encodeproject.org/files/ENCFF957FQF/@@download/ENCFF957FQF.bigWig
https://www.encodeproject.org/files/ENCFF464YSK/@@download/ENCFF464YSK.bigWig

https://www.encodeproject.org/files/ENCFF834HNV/@@download/ENCFF834HNV.bigWig
https://www.encodeproject.org/files/ENCFF801HPN/@@download/ENCFF801HPN.bigWig
https://www.encodeproject.org/files/ENCFF062LIE/@@download/ENCFF062LIE.bigWig
https://www.encodeproject.org/files/ENCFF328UMQ/@@download/ENCFF328UMQ.bigWig
https://www.encodeproject.org/files/ENCFF606CWZ/@@download/ENCFF606CWZ.bigWig
https://www.encodeproject.org/files/ENCFF923FFS/@@download/ENCFF923FFS.bigWig

https://www.encodeproject.org/files/ENCFF038HNR/@@download/ENCFF038HNR.bigWig
https://www.encodeproject.org/files/ENCFF388WMD/@@download/ENCFF388WMD.bigWig
https://www.encodeproject.org/files/ENCFF981WTU/@@download/ENCFF981WTU.bigWig
https://www.encodeproject.org/files/ENCFF958BAN/@@download/ENCFF958BAN.bigWig
https://www.encodeproject.org/files/ENCFF420DLT/@@download/ENCFF420DLT.bigWig

FPKM was computed using transcript quantification from the following ENCODE data links:
https://www.encodeproject.org/files/ENCFF091UPQ/@@download/ENCFF091UPQ.tsv
https://www.encodeproject.org/files/ENCFF705JDM/@@download/ENCFF705JDM.tsv
https://www.encodeproject.org/files/ENCFF138HMO/@@download/ENCFF138HMO.tsv
https://www.encodeproject.org/files/ENCFF855ALP/@@download/ENCFF855ALP.tsv

Repli-Seq data was downloaded from the following accessions:
GM12878: GSM923451
K562: GSM923448
IMR90: GSM923447
HeLa: GSM923449
HUVEC: GSM923452

TSA-seq data was downloaded from GSE66019

Field-specific reporting

Please select the one below that is the best fit for your research. If you are not sure, read the appropriate sections before making your selection.

☒ Life sciences ☐ Behavioural & social sciences ☐ Ecological, evolutionary & environmental sciences

For a reference copy of the document with all sections, see nature.com/documents/nr-reporting-summary-flat.pdf

Life sciences study design

All studies must disclose on these points even when the disclosure is negative.

Sample size	Sample sizes were not computed because the dimensions of the inter-chromosomal Hi-C matrix is fixed. The sample size was chosen based on the number of 100kb bins in the human genome. It is sufficient for the training of our model because there are over 26,000 mapped 100kb bins in the human genome.
Data exclusions	Not applicable
Replication	We re-trained models from scratch on a completely new python environment, and we found that all attempts at replication were successful - the vast majority of predictions matched our original results.
Randomization	Samples were divided such that the model was trained on genomic bins occupying approximately half of the genome and tested on the remaining genomic bins. This allocation was not random and we controlled covariates by constructing our training and test sets using genomic bins in only a subset of chromosomes. We also performed 10-fold cross validation in which training samples were chosen randomly, although the final model was based on the non-random allocation.

Blinding	This division mentioned above makes our models blind to chromosomes not in the training set and serves to assess the bias of our model towards chromosomes that it is not blind to.

Reporting for specific materials, systems and methods

We require information from authors about some types of materials, experimental systems and methods used in many studies. Here, indicate whether each material, system or method listed is relevant to your study. If you are not sure if a list item applies to your research, read the appropriate section before selecting a response.

Materials & experimental systems

n/a	Involved in the study
☒	☐ Antibodies
☒	☐ Eukaryotic cell lines
☒	☐ Palaeontology
☒	☐ Animals and other organisms
☒	☐ Human research participants
☒	☐ Clinical data

Methods

n/a	Involved in the study
☒	☐ ChIP-seq
☒	☐ Flow cytometry
☒	☐ MRI-based neuroimaging
